# Activation of Lysosomal Retrograde Transport Triggers TPC1‐IP3R1 Ca^2+^ Crosstalk at Lysosome‐ER MCSs Leading to Lethal Depleting of ER Calcium

**DOI:** 10.1002/advs.202415313

**Published:** 2025-07-25

**Authors:** Meng‐yuan Zhu, Yong‐jian Guo, Yu‐qi Zhu, Hong‐zheng Wang, Hai‐di Wang, Hong‐yu Chen, Yue‐xin Jiang, Hui Li, Hui Hui

**Affiliations:** ^1^ State Key Laboratory of Natural Medicines Jiangsu Key Laboratory of Carcinogenesis and Intervention Key Laboratory of Drug Quality Control and Pharmacovigilance Ministry of Education Jiangsu Key Laboratory of Drug Design and Optimization China Pharmaceutical University 24 Tongjiaxiang Nanjing 210009 P. R. China; ^2^ Bayi Hospital Affiliated to Nanjing University of Chinese Medicine 138 Xianlin Rd Nanjing 210023 P. R. China

**Keywords:** calcium crosstalk, ERS, LIMP2, lysosomal dynamics, membrane contact site, organelle interaction

## Abstract

Inter‐organellar signaling linkages in oncology are increasingly elucidated. However, the impact of lysosome‐endoplasmic reticulum (ER) interaction on tumor cell fate remains relatively unexplored. A novel interaction between lysosomes and the ER, mediated by the flavonoid LW‐213 through targeting LIMP2 (lysosomal integral membrane protein type 2)to activate a lysosomal repair pathway, is identified in acute myeloid leukemia (AML). This leads to activated RAB7A activity, enhancing lysosomal retrograde transport to the perinuclear region and increasing contact at lysosome‐ER membrane contact sites (MCSs). Close proximity of TPC1 to IP3R1 at these sites generates a concentrated calcium microdomain, triggering Ca^2+^‐induced Ca^2+^ release, which causes cytoplasmic calcium turbulence and two distinct calcium tides. This excessive calcium efflux depletes ER calcium stores, triggering lethal ER stress‐induced apoptosis. Interestingly, altering TPC1 expression levels in HeLa cells affected these calcium dynamics, replicating AML‐specific mechanisms when overexpressed. Subsequent studies using BALB/c xenograft models with wild‐type and LIMP2‐knockout THP1 cells, along with ICR mice toxicity models, confirmed LW‐213′s significant tumor growth inhibition with minimal toxicity. These findings underscore the potential of targeting lysosomal‐ER calcium crosstalk as an innovative approach to cancer treatment, highlighting the therapeutic promise of LW‐213 in managing tumor cell fate through modulating organellar interactions.

## Graphical abstract

The flavonoid compound LW‐213 exerts its antitumor effects through a novel mechanism involving lysosomal‐ER calcium regulation. Specifically, LW‐213 binds to LIMP2, which induces perinuclear clustering of lysosomes and facilitates calcium‐mediated lysosome‐ER interactions. This molecular interaction triggers endoplasmic reticulum calcium depletion, subsequently activating the ER stress pathway and ultimately inducing apoptosis in tumor cells.

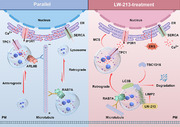

## 1 Introduction

A fundamental characteristic of eukaryotic cells is their membrane‐bound compartments, which isolate biochemical processes spatially and temporally.^[^ ^1^, ^2^, ^3^ ^]^ Recent studies indicate that organelles form dynamic networks, rather than static entities, facilitating communication through MCSs without undergoing fusion.^[^ ^3^, ^4^, ^5^ ^]^

MCSs enable the transfer of molecular and informational signals between organelles.^[^ ^6^ ^]^ The structural and signaling dynamics of MCSs vary, with growing evidence underscoring the crucial role of ER‐based MCSs in regulating calcium signaling across compartments.^[^ ^7^, ^8^ ^]^ The ER serves as the primary intracellular calcium store and regulates Ca^2+^ balance through the SERCA pump and IP3R1 channel.^[^ ^9^ ^]^ Store‐operated Ca^2+^ entry (SOCE) is activated by sensing a reduction in ER Ca^2+^ levels and facilitates the influx of extracellular Ca^2+^ through the essential components stromal interaction molecule 1(STIM1) in the ER and ORAI calcium release‐activated calcium modulator 1(ORAI1) in the plasma membrane.^[^ ^10^ ^]^ The formation of MCSs between the ER and the plasma membrane triggers structural alterations that activate ORAI1 channels for extracellular Ca^2^⁺ entry following ER Ca^2^⁺ depletion.^[^ ^11^, ^12^, ^13^ ^]^ Related studies indicate that the ER calcium store serves as a primary source for the lysosomal calcium pool. Through homeostatic regulation at MCSs, it controls lysosomal calcium homeostasis, thereby ensuring the proper execution of lysosome‐mediated biological processes. The reciprocal calcium exchange between lysosomes and the ER involves counteractive processes where lysosomal calcium release impacts the ER within specific microdomains, allowing critical signals to be enhanced by ER‐based amplifiers.^[^ ^14^, ^15^, ^16^, ^17^, ^18^ ^]^ This localized surge in Ca^2+^ initiates a positive feedback mechanism known as Ca^2+^‐induced Ca^2+^ release (CICR).^[^ ^19^, ^20^, ^21^ ^]^

The lysosomal calcium pool is critical for cellular biological processes. Lysosomal calcium release channels are a specialized group of proteins located on the lysosomal membrane, responsible for regulating the release of Ca^2+^ from within the lysosome to the cytoplasm.^[^ ^22^ ^]^ These channels include the Two‐Pore Channel (TPC) family, such as TPC1 and TPC2, as well as the Transient Receptor Potential Mucolipin (TRPML) family, among others. They play a pivotal role in intracellular calcium signaling, the maintenance of lysosomal function, and cellular metabolic processes. For instance, the TPC1 channel can respond to signals from Nicotinic Acid Adenine Dinucleotide Phosphate (NAADP), thereby opening the channel to allow Ca^2+^ efflux, influencing processes such as autophagy, membrane repair, and signal transduction in cells.^[^ ^23^ ^]^ Meanwhile, the TRPML1 channel is closely associated with lysosomal acidification, material transport, and autophagosome maturation.^[^ ^24^ ^]^ Dysfunctions in lysosomal calcium release channels are linked to various diseases, including neurodegenerative disorders, lysosomal storage diseases, and cancer.^[^ ^25^ ^]^ Consequently, they have become significant subjects in biomedical research, offering new perspectives and strategies for the diagnosis and treatment of diseases.

The establishment of MCSs is dependent on the ability of organelles to traverse considerable distances within the cytoplasm and along microtubules, a process aided by adapter proteins that link with microtubule motors.^[^ ^26^ ^]^ Lysosomal positioning within the cell is largely influenced by bidirectional microtubule‐dependent transport, supported by small GTPases that bind to the lysosomal cytoplasmic surface and effectors that interact with kinase or motor proteins. For instance, the ARL8b GTPase promotes the forward movement of lysosomes toward the cell periphery via its effector SKIP, while RAB7 GTPase enhances reverse transport through its interaction with RILP.^[^ ^27^, ^28^, ^29^ ^]^ GTPases located on lysosomal membranes are categorized into five distinct subfamilies: Ras, Rho, Rab, Arf/Sar, and Ran. Within the Ras‐like superfamily, Rab protein's function as molecular switches, alternating between active (GTP‐bound) and inactive (GDP‐bound) states. This dynamic cycling is tightly regulated by GTPase activating proteins (GAPs) and guanine nucleotide exchange factors (GEFs), which govern the activation and deactivation of GTPase activity. Rab GAPs accelerate GTP hydrolysis to deactivate Rab proteins.^[^ ^30^, ^31^ ^]^ Among these, RAB7A predominantly manages transport from early endosomes to late endosomes and then to lysosomes;^[^ ^32^ ^]^ in contrast, RAB7B adjusts transport from lysosomes to the Golgi apparatus.^[^ ^33^ ^]^

Lysosomal membrane‐integrated protein 2 (LIMP2), also identified as scavenger receptor family B2 (SCARB2), plays a vital role in regulating lipid metabolism and innate immunity.^[^ ^34^ ^]^ Studies have shown that it assists in transporting the acid hydrolase β‐glucosidase from the ER to lysosomes through its pore‐like structure at the end of a helical coil.^[^ ^35^, ^36^, ^37^ ^]^ Additionally, its extracellular domain features a spacious cavity that aids in the transfer of cholesterol and possibly other lipids to the lysosomal membranes, contributing to lipid transportation.^[^ ^38^ ^]^ Recent research has linked LIMP2 with autophagy; it promotes autolysosome formation, thereby enhancing autophagic flux and affects tumor cell proliferation by modulating stem‐like properties.^[^ ^39^ ^]^

TBC1D15, a member of the TBC domain family, negatively regulates the Rab family of small GTPases by hydrolyzing lysosomal RAB7.^[^ ^40^ ^]^ It plays a role in controlling lysosomal transport, fusion, and maturation.^[^ ^41^ ^]^ Mammalian Fis1 recruits TBC1D15 to regulate mitochondrial morphology and support the formation of mitochondrial‐lysosomal MCSs.^[^ ^42^, ^43^ ^]^ Additionally, TBC1D15 can be recruited directly by lysosomes. Acute lysosomal membrane damage diminishes the number of operational lysosomes within cells; however, these lysosomes possess an inherent recovery capability that is unaffected by the depletion of TFEB or TFE3. This delineates a pathway for lysosomal membrane regeneration, involving LC3B, LIMP2, RAB7A GTPase negative regulator TBC1D15, and proteins essential for autophagosome assembly (including Dynamin‐2, Kinesin‐5B, and Clathrin). Upon lysosomal damage, LIMP2 serves as a receptor by binding to LC3B; TBC1D15 interacts with LC3B on damaged lysosomes and provides a framework for assembling and stabilizing the autophagosome assembly machinery, thereby promoting the formation of lysosomal tubules and subsequent Dynamin‐2‐dependent division.^[^ ^44^ ^]^ In this scenario, the LIMP2‐LC3B‐TBC1D15 complex supports a novel mechanism of lysosomal regeneration.

In this study, we utilized the flavonoid compound LW‐213 to target LIMP2, enhancing the formation of the LIMP2‐LC3B‐TBC1D15 heterotrimer to activate lysosomal degradation of TBC1D15. This process alleviated the inhibition of RAB7 activity, enhanced lysosomal retrograde transport, and facilitated increased MCSs formation between lysosomes and the ER. The unique inter‐organelle “nanodomain” structure established a robust foundation for subsequent calcium signaling events. We demonstrated that reduced ER calcium capacity, triggered by lysosomal TPC1 calcium signals, induced ER stress and apoptosis through the ER stress pathway. Additionally, we found that this anti‐tumor strategy is selective and exhibits enhanced anti‐tumor effects specifically in AML with high TPC1 expression levels.

## 2 Results

### 2.1 Relevance of TPCN1 Gene Expression in Determining the Efficacy of LW‐213

**Figure 1:**
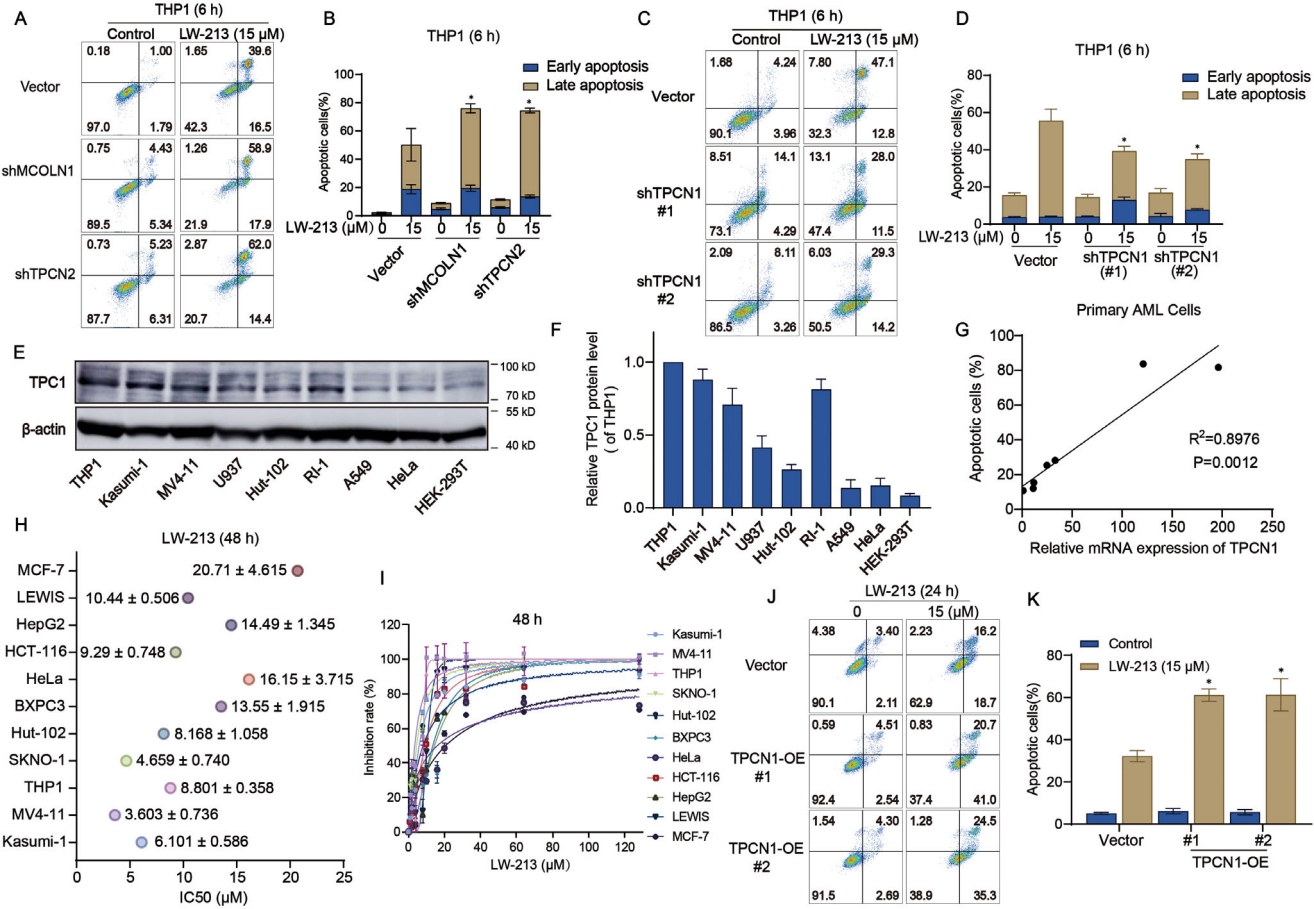
Relevance of TPCN1 gene expression in determining the efficacy of LW‐213. A,B) Flow cytometric analysis of Annexin V‐FITC/PI‐PerCP‐stained cell lines of THP1 or THP1 with TRPML1 (Gene Symbol: MCOLN1) and TPC2 (Gene Symbol : TPCN2) knockdown treated with 15 µm of LW‐213 for 6 h, ^*^ *p* < 0.05 compared to Vector 15 µm group. (C‐D) Flow cytometric analysis of Annexin V‐FITC/PI‐PerCP‐stained cell lines of THP1 or THP1 with TPC1 (Gene Symbol: TPCN1) knockdown (#1, #2) treated with 15 µm of LW‐213 for 6 h, ^*^ *p* < 0.05 compared to Vector 15 µm group. E,F) The expression of TPC1 protein was assessed by extracting total proteins from various tumor cell lines and HEK 293T cells, β‐actin was used as a loading control. G) GraphPad Prism 9.0 was used to generate a correlation plot between TPCN1 gene expression and LW‐213‐induced apoptosis. The coefficient of determination (R^2^) is commonly used to assess regression model quality, the closer R^2^ is to 1, the better the fit of the equation. p<0.05 indicated significant differences. H,I) The growth inhibition effect of LW‐213 on various tumor cell lines were assessed by CCK8 assay at 48 h. J,K) Flow cytometric analysis of Annexin V‐FITC/PI‐PerCP‐stained cell lines of HeLa or HeLa with TPCN1 overexpression (#1, #2) treated with 15 µm of LW‐213 for 24 h, ^*^ *p* < 0.05 compared to Vector 15 µm group. Data are shown as Mean ± S.E.M. from three independent experiments. ^*^ *p* < 0.05, ^**^ *p* < 0.01, and ^***^ *p* < 0.001, ns indicates non‐significant.

Previous studies have shown that LW‐213, a derivative of wogonin, targets lysosomes in T‐cell lymphoma due to its mildly alkaline properties and exerts a significant antitumor effect. A critical component in this mechanism involves calcium signaling through the lysosomal TRPML1 channel.^[^ ^45^, ^46^ ^]^ Notably, LW‐213 induced apoptosis in AML cell lines, achieving effects comparable to or surpassing those in T‐cell lymphoma. Building on this foundation, our study initially explored lysosomal calcium channels as potential targets. Individual knockdown experiments of TRPML1, TPC1, and TPC2 (Ca^2+^ release channels in lysosomes) in THP1 cells revealed a notable reduction in apoptosis with TPC1 knockdown (**Figure** **1**A–D; Figure S1A,B, Supporting Information). Ned‐19,^[^ ^16^, ^47^ ^]^ identified as a specific inhibitor of calcium signal release from TPC1, was used in co‐treatment experiments with LW‐213 for 6 h, showing an inhibition of apoptosis (Figure S1C,D, Supporting Information). These observations support the critical role of TPC1 in mediating the anti‐leukemia effects of LW‐213. The TCGA database indicated a significantly higher expression of TPC1 in AML compared to other tumors (Figure S1E, Supporting Information). We measured mRNA and protein levels of TPC1 in various tumor cells, finding elevated levels in all tested AML cell lines (Figure 1E,F; Figure S1F, Supporting Information). To further establish the strong dependence of LW‐213′s anti‐AML effects on TPCN1, we analyzed seven clinical samples from AML patients (Table S1, Supporting Information). The correlation analysis showed that patients with high TPCN1 expression responded better to LW‐213′s apoptotic effects (Figure 1G). Conversely, LW‐213 was less effective in inhibiting proliferation and inducing apoptosis in other tumor cells with lower TPCN1 expression (Figure 1H,I, Figure S1G, Supporting Information). Assuming a positive correlation between LW‐213 efficacy and TPCN1 expression, overexpressing TPCN1 in cells with initially low levels enhanced the apoptotic response to LW‐213 treatment. In experiments with HeLa cells, which naturally express low TPCN1, overexpression led to a marked increase in apoptosis compared to the control group (Figure 1J,K; Figure S1H–L, Supporting Information). This evidence compellingly demonstrates that TPCN1 levels significantly influence tumor behavior and progression, ultimately determining the antitumor efficacy of LW‐213.

### 2.2 Ca^2 +^‐Mediated Dialogue between Lysosome and ER at MCSs

**Figure 2:**
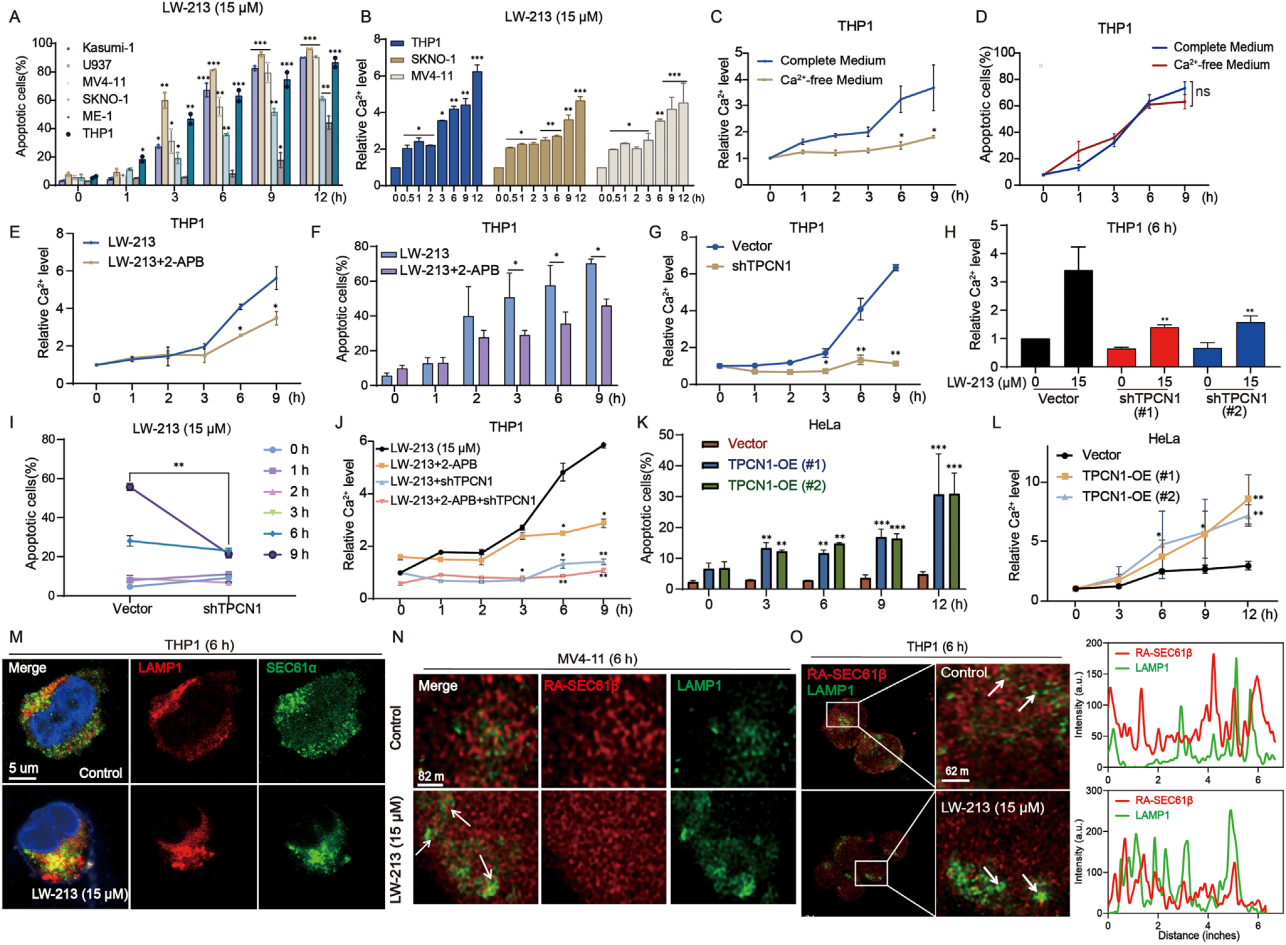
Ca^2 +^‐mediated dialogue between lysosome and ER at MCSs. A) Flow cytometric analysis of Annexin V‐FITC/PI‐PerCP‐stained cell lines of Kasumi‐1, U937, MV4‐11, SKNO‐1, ME‐1, and THP1 treated with 15 µm of LW‐213 for 1, 3, 6, 9, and 12 h., ^*^ *p* < 0.05, ^**^ *p* < 0.01, ^***^ *p* < 0.001 compared to 0 h group. B) The THP1, SKNO‐1 and MV4‐11 cells were exposed to 15 µM of LW‐213 for 0.5, 1, 2, 3, 6, 9, and 12 h, respectively. The Ca^2+^ indicator Fluo3‐AM measured cytoplasmic Ca^2+^ levels in each group of cells, ^*^ *p* < 0.05, ^**^ *p* < 0.01, and ^***^ *p* < 0.001 compared to 0 h group. C) The THP1 cells were exposed to 15 µM of LW‐213 with complete medium or Ca^2+^‐free medium for 1, 2, 3, 6, and 9 h, respectively. The Ca^2+^ indicator Fluo3‐AM measured cytoplasmic Ca^2+^ levels in each group of cells, ^*^ *p* < 0.05, ^**^ *p* < 0.01, ^***^ *p* < 0.001 compared to Complete Medium group. D) Flow cytometric analysis of Annexin V‐FITC/PI‐PerCP‐stained cell line of THP1 treated with 15 µm of LW‐213 with complete medium or Ca^2+^‐free medium for 1, 3, 6, and 9 h, ns indicates non‐significant compared to Complete Medium group. E) The THP1 cells were pretreated with 2‐APB (100 µM) for 2 h, then exposed to 15 µm LW‐213 for 1, 2, 3, 6, and 9 h. The Ca^2+^ indicator Fluo3‐AM measured cytoplasmic Ca^2+^ levels in each group of cells, ^*^ *p* < 0.05 compared to LW‐213 group. F) Flow cytometric analysis of Annexin V‐FITC/PI‐PerCP‐stained cell line of THP1 treated with 15 µm of LW‐213 with/without 2‐APB (100 µm) for 1, 2, 3, 6, and 9 h, ^*^ *p* < 0.05 compared to LW‐213 group. G) The Vector and shTPCN1 THP1 cells were exposed to 15 µm LW‐213 for 1, 2, 3, 6, and 9 h, respectively. The Ca^2+^ indicator Fluo3‐AM measured cytoplasmic Ca^2+^ levels in each group of cells, ^*^ *p* < 0.05, ^**^ *p* < 0.01 compared to Vector group. H) The Vector and shTPCN1 THP1 cells were exposed to 15 µm LW‐213 for 6 h, respectively. The Ca^2+^ indicator Fluo3‐AM measured cytoplasmic Ca^2+^ levels in each group of cells, ^**^ *p* < 0.01 compared to Vector 15 µm LW‐213 group. I) Flow cytometric analysis of Annexin V‐FITC/PI‐PerCP‐stained cell lines of Vector or shTPCN1 THP1 treated with 15 µm of LW‐213 for 1, 2, 3, 6, and 9 h, ^**^ *p* < 0.01 compared to Vector 15 µm LW‐213 group. J) The Vector and shTPCN1 THP1 cells were exposed to 15 µm LW‐213 with/without 2‐APB (100 µm) for 1, 2, 3, 6, and 9 h, respectively. The Ca^2+^ indicator Fluo3‐AM measured cytoplasmic Ca^2+^ levels in each group of cells, ^*^ *p* < 0.05, ^**^ *p* < 0.01 compared to 15 µm LW‐213 group. K) Flow cytometric analysis of Annexin V‐FITC/PI‐PerCP‐stained cell lines of Vector or TPCN1‐OE (#1, #2) HeLa treated with 15 µm of LW‐213 for 3, 6, 9, and 12 h, ^**^ *p* < 0.01, ^***^ *p* < 0.001 compared to Vector group.(L) The Vector and TPCN1‐OE (#1, #2) HeLa treated with 15 µM of LW‐213 for 3, 6, 9, and 12 h, respectively. The Ca^2+^ indicator Fluo3‐AM measured cytoplasmic Ca^2+^ levels in each group of cells, ^*^ *p* < 0.05, ^**^ *p* < 0.01 compared to Vector group. M) The THP1 cells were treated with 15 µm of LW‐213 for 6 h. Cells were collected and crawled for immunofluorescence staining of cell nuclei for DAPI (blue), LAMP1 protein (red), and SEC61α (green). They were detected by confocal microscopy (FV1000; Olympus) with FV10‐ASW2.1 acquisition software (Olympus) at room temperature (original magnification × 1000; immersion objective × 100 × 40 with immersion oil type) (total cells in each group >100). N,O) THP1 and MV4‐11 cells transfected with RA‐SEC61β were treated with 15 µm of LW‐213 for 6 h. Cells were collected and crawled for immunofluorescence staining of LAMP1 protein (green). They were detected by confocal microscopy (Leica Ultra‐High‐Resolution Laser Confocal) at room temperature (original magnification × 1000; immersion objective × 100 × 40 with immersion oil type) (total cells in each group >100). Data are shown as Mean ± S.E.M. from three independent experiments. ^*^ *p* < 0.05, ^**^ *p* < 0.01, ^***^ *p* < 0.001, ns indicates non‐significant.

Next, the role of TPCN1 in the anti‐tumor efficacy of LW‐213 will be further investigated. Previous research has established a close association between LW‐213′s effect and calcium signaling. To explore this mechanism further, six AML cell lines were monitored dynamically for 12 h after treatment with 15 µm LW‐213. The results showed that LW‐213 induced apoptosis in these cell lines (**Figure** **2**A; Figure S2A, Supporting Information). Concurrently, cytoplasmic Ca^2+^ levels showed two distinct fluctuations, divided by a point at 6 h (Figure 2B). The initial phase showed a slight elevation of cytoplasmic Ca^2+^ levels, termed the initial “calcium tide,” followed by a significant increase, the secondary “calcium tide”. Live cell imaging in THP1 cells treated with LW‐213 for 9 h confirmed these stages (Video S1, Supporting Information). An initial experiment using a Ca^2+^‐free medium to limit exogenous Ca^2+^ uptake showed a significant drop in cytoplasmic Ca^2+^ levels (Figure 2C), yet this did not markedly alter the apoptotic impact of LW‐213 (Figure 2D; Figure S2B, Supporting Information). This suggests that LW‐213‐induced apoptosis might rely on endogenous Ca^2+^ release. The inhibition of IP3R1 with 2‐APB in THP1 cells led to a significant reduction in the secondary wave of cytoplasmic Ca^2+^ (Figure 2E) and decreased the apoptotic effects of LW‐213 (Figure 2F; Figure S2C,D, Supporting Information). This indicates that IP3R1 plays a crucial role in LW‐213‐induced apoptosis. However, given the incomplete suppression of effects upon IP3R1 inhibition and considering LW‐213′s association with the TPC1 lysosomal Ca^2+^ release pathway, it is tentatively concluded that both TPC1 and IP3R1 cooperatively regulate the increase in cytoplasmic Ca^2+^.

To test the hypothesis, real‐time monitoring of cytoplasmic Ca^2+^ dynamics in THP1 cells was conducted following TPC1 knockdown and treatment with 15 µm LW‐213. The results showed that suppression of TPC1 Ca^2+^ signaling effectively abolished the Ca^2+^ oscillations induced by LW‐213 (Figure 2G,H). Knockdown of TPC1 restored cytoplasmic Ca^2+^ to basal levels and significantly attenuated the apoptosis induced by LW‐213 (Figure 2I; Figure S2E, Supporting Information). A comparison of cytoplasmic Ca^2+^ levels upon interference with TPC1 and IP3R1 revealed that inhibition of IP3R1 alone only reduced the second phase of cytoplasmic Ca^2+^. However, inhibition of TPC1 Ca^2+^ signaling eliminated both waves of “calcium tides” induced by LW‐213, indicating simultaneous inhibition of both channels (Figure 2J). Thus, it was established that lysosomal TPC1 Ca^2+^ signaling precedes IP3R1 signaling. Consistent with previous findings, LW‐213 did not induce sharp cytoplasmic Ca^2+^ oscillations in HeLa cells expressing low levels of TPC1 (Figure S2F, Supporting Information), whereas overexpression of TPC1 increased the sensitivity of HeLa cells to LW‐213 (Figure 2K) and elevated intracellular Ca^2+^ levels (Figure 2L), suggesting that the presence of TPC1 facilitates Ca^2+^ communication across organelles. TPC1 and IP3R1 must be closely positioned to establish MCSs for Ca^2+^ exchange. To validate this hypothesis, immunofluorescence assays were performed on THP1 cells treated with 15 µm LW‐213 for 6 h. These assays demonstrated significant co‐localization between lysosomes (labeled with LAMP1) and the ER (labeled with SEC61α) (Figure 2M). High‐resolution images showed lysosomes, initially dispersed, being mobilized by LW‐213, leading to noticeable co‐localization and an increase in MCSs between the ER (labeled with RA‐SEC61β plasmids) and lysosomes (Figure 2N,O). Moreover, under the influence of LW‐213, live cell imaging captured prolonged co‐localization of lysosomes and the ER (Video S2, Supporting Information). In conclusion, TPC1 regulated IP3R1‐mediated Ca^2+^ release through MCSs, where Ca^2+^ released by TPC1 acts as “calcium sparks” within a confined space; this initiates a positive feedback loop via CICR that activates IP3R1, sensitive to Ca^2+^, thereby facilitating further release of Ca^2+^ from IP3R1.

### 2.3 ER Calcium Depletion Triggered by TPC1 is Directly Responsible for LW‐213‐Induced Apoptosis

**Figure 3:**
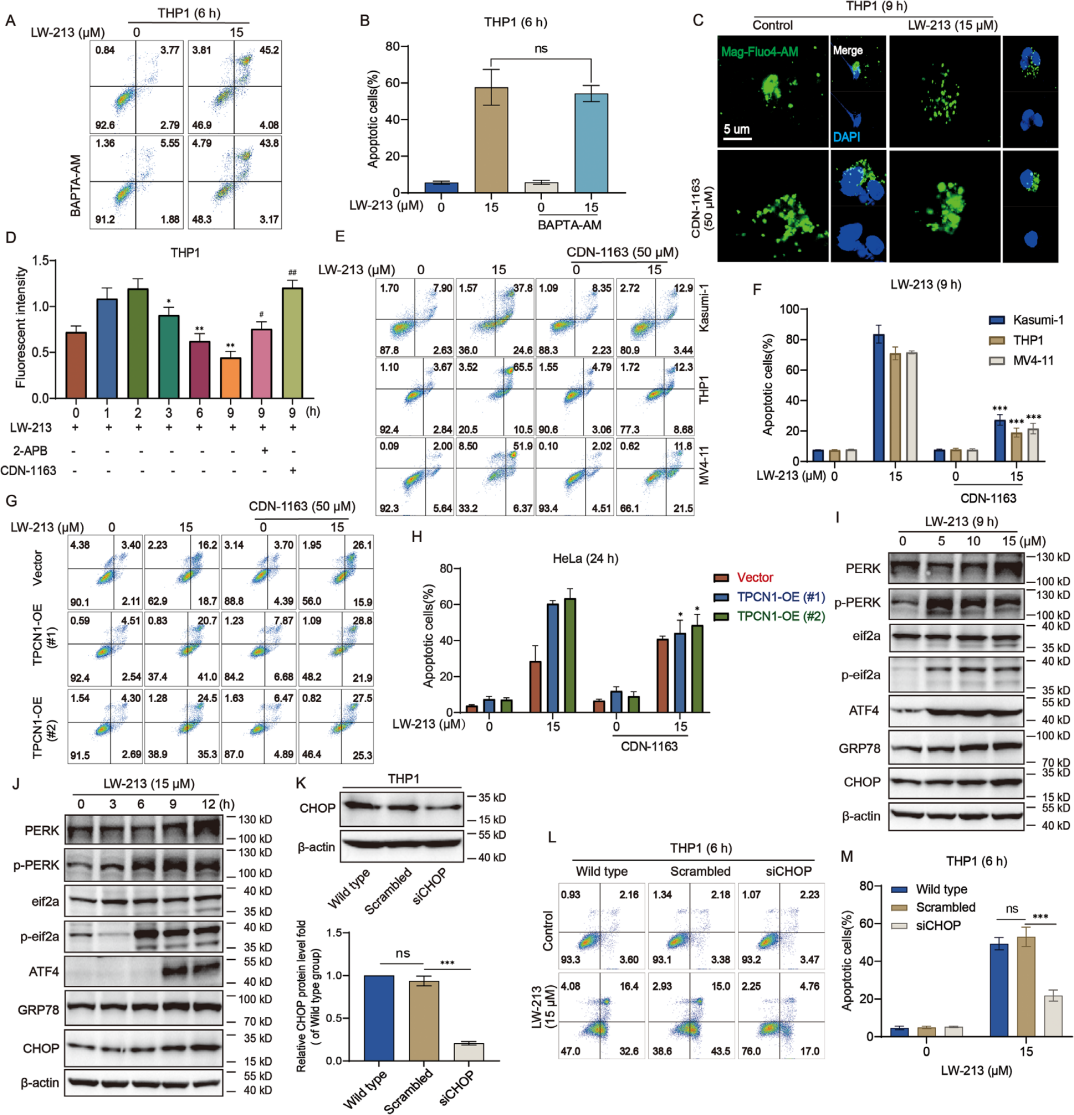
ER calcium depletion triggered by TPC1 is directly responsible for LW‐213‐induced apoptosis. A,B) Flow cytometric analysis of Annexin V‐FITC/PI‐PerCP‐stained cell line of THP1 treated with 15 µm of LW‐213 with/without BAPTA‐AM (10 µm) for 6 h, ns compared to 15 µm LW‐213 group. C) The THP1 cells were treated with 15 µm of LW‐213 with/without CDN‐1163 (50 µm) for 9 h. Cells were collected and crawled for immunofluorescence staining of cell nuclei for DAPI (blue), Mag‐Fluo4‐AM (green). They were detected by confocal microscopy (FV1000; Olympus) with FV10‐ASW2.1 acquisition software (Olympus) at room temperature (original magnification × 1000; immersion objective × 100 × 40 with immersion oil type) (total cells in each group >100). (D) Flow cytometric analysis of Mag‐Fluo4‐AM‐stained cell line of THP1 treated with 15 µm of LW‐213 with/without 2‐APB (100 µm) or CDN‐1163 (50 µM) for 1, 2, 3, 6, and 9 h, ^*^ *p* < 0.05, ^**^ *p* < 0.01 compared to 0 h LW‐213 group, ^#^ *p* < 0.05, ^##^ *p* < 0.01 compared to 9 h LW‐213 group. E,F) Flow cytometric analysis of Annexin V‐FITC/PI‐PerCP‐stained cell line of THP1 treated with 15 µM of LW‐213 with/without CDN‐1163 (50 µm) for 9 h, ^***^ *p* < 0.001 compared to 15 µm LW‐213 group. (G‐H) Flow cytometric analysis of Annexin V‐FITC/PI‐PerCP‐stained cell line of Vector or TPCN1‐OE HeLa treated with 15 µm of LW‐213 with/without CDN‐1163 (50 µm) for 24 h, ^*^ *p* < 0.05 compared to 15 µm LW‐213 group. I) The THP1 cells were exposed to LW‐213 (5, 10, and 15 µm) for 9 h. The expression levels of ERS proteins were analyzed by western blot. β‐actin was used as a loading control. J) The THP1 cells were exposed to LW‐213 (15 µm) for 3, 6, 9, and 12 h. The expression levels of ERS proteins were analyzed by western blot. β‐actin was used as a loading control. K) CHOP knockdown efficiency in THP1 cells. β‐actin was used as a loading control, ns indicates non‐significant compared to wild type group, ^***^ *p* < 0.001 compared to Scrambled group. L,M) Flow cytometric analysis of Annexin V‐FITC/PI‐PerCP‐stained cell line of THP1 and siCHOP‐THP1 treated with 15 µmfigure of LW‐213 for 6 h, ns indicates non‐significant compared to wild type group, ^***^ *p* < 0.001 compared to Scrambled group. Data are shown as Mean ± S.E.M. from three independent experiments. ^*^ *p* < 0.05, ^**^ *p* < 0.01, ^***^ *p* < 0.001, ns indicates non‐significant.

Our study has established that TPC1 and IP3R1 collaboratively mediate cytoplasmic Ca^2+^ oscillation accompanied by apoptosis. However, determining a direct causal relationship between these events is crucial for developing novel anti‐tumor strategies. Initially, our aim was to determine whether elevated cytoplasmic Ca^2+^ directly induces apoptosis. To block the effects of cytoplasmic Ca^2+^, we treated cells with LW‐213 and the Ca^2+^ chelator BAPTA‐AM for 6 h. Annexin V‐PI staining showed that chelation of elevated intracellular Ca^2+^ does not prevent LW‐213‐induced apoptosis (Figure **3**A,B), suggesting that cytoplasmic Ca^2+^ increase does not initiate apoptosis. If free Ca^2+^ does not trigger apoptosis, then the organelle releasing this Ca^2+^ could provide an alternative mechanism. As a key factor in the second wave of the “calcium tide,” a significant release of Ca^2+^ from IP3R1 and reduced Ca^2+^ storage in the ER might induce apoptosis through ER stress. Our research focused on the ER, using Mag‐Fluo4‐AM as a specific probe for its Ca^2+^. Confocal fluorescence imaging showed that LW‐213 treatment resulted in a decrease in Mag‐Fluo4‐AM fluorescence intensity, indicating a lower Ca^2+^ capacity in the ER (Figure 3C). Typically, maintaining Ca^2+^ storage equilibrium depends primarily on IP3R1 and SERCA. The primary mechanisms reducing capacity include activating IP3R1‐mediated release and inhibiting SERCA‐mediated uptake. We analyzed SERCA function under LW‐213 treatment by assessing its activity and protein expression, finding no impact from the treatment (Figure S3A–F, Supporting Information).

SERCA inhibition was excluded, resulting in reduced Ca^2+^ storage within the ER, and the excessive release of Ca^2+^ from IP3R1 emerged as the main focus of this study. Dynamic monitoring of ER Ca^2+^ in THP1 cells utilized an ER Ca^2+^ indicator probe for 9 h of LW‐213 treatment. Within the first 2 h, a compensatory increase in ER Ca^2+^ storage was observed, correlating with the typical initiation of SOCE. Immunofluorescence results indicated that STIM1 aggregation increased as the duration of LW‐213 treatment extended, with BHQ acting as a positive control^[^ ^48^ ^]^ (Figure 3D; Figure S3G–I, Supporting Information). This was due to the ER responding to signals of Ca^2+^ depletion by inputting Ca^2+^ via SOCE and replenishing it with SERCA pumps.^[^ ^49^ ^]^ These results suggested the preserved functionality of the ER calcium pump. From 3 h onwards, a time‐dependent reduction in ER calcium capacity occurred, and was significantly reversed by using 2‐APB and the SERCA agonist CDN‐1163 (Figure 3C,D), leading to decreased apoptosis in the AML cell line (Figure 3E,F). Furthermore, once Ca^2+^ levels within the ER recovered (Figure S3I,J, Supporting Information), SOCE induced by decreased ER Ca^2+^ storage ceased.

To further establish the direct role of TPC1 Ca^2+^ signaling in triggering extensive IP3R1 Ca^2+^ release, HeLa cells transfected with empty vector and TPCN1‐overexpressing plasmids were simultaneously treated with LW‐213 for 24 h. Apoptosis data showed a notable decrease in TPCN1‐overexpressing HeLa cells following treatment with CDN‐1163, while no change was noted in the empty vector group (Figure 3G,H). These results strongly supported the hypothesis that TPC1 Ca^2+^ signaling contributes to ER Ca^2+^ capacity reduction. Crucially, reduced ER Ca^2+^ was linked with apoptosis via ER stress, as evidenced by the time‐ and concentration‐dependent increase in PERK‐eif2a‐ATF4‐CHOP signaling pathway (Figure 3I,J; Figure S3K,L, Supporting Information). Meanwhile, knocking down the expression of CHOP could significantly reduce the apoptotic efficacy of LW‐213 (Figure 3K–M). In summary, localized TPC1 Ca^2+^ signaling at lysosomal‐ER MCSs acted through an “amplifier” mechanism involving IP3R1, leading to reduced ER Ca^2+^ storage and ultimately facilitating tumor cell apoptosis via the ER stress pathway.

### 2.4 Augmented Retrograde Transport of Lysosomes Enhances Ca^2+^ Crosstalk

**Figure 4:**
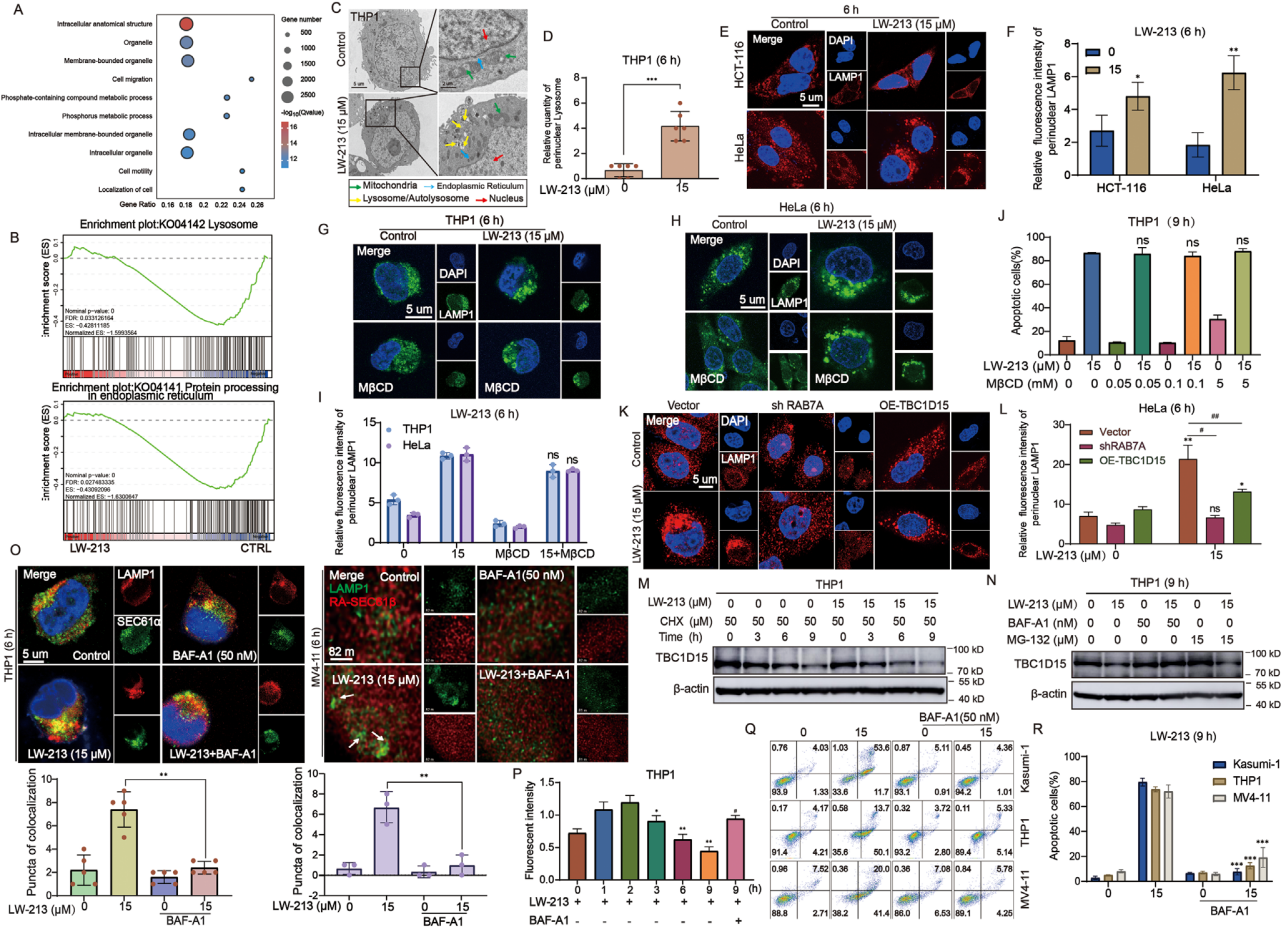
Augmented retrograde transport of lysosomes enhances Ca^2+^ crosstalk. A,B) The THP1 cells were exposed to LW‐213 (15 µm) for 6 h. The Total RNA was extracted from the cells for RNA‐seq analysis. C,D) The THP1 cells were exposed to LW‐213 (15 µm) for 6 h. The distribution of lysosomes was examined by bio‐transmission electron microscopy, as indicated by the yellow arrow in the Figure, ^***^ *p* < 0.001 compared to 0 µm LW‐213 group. E,F) The HCT‐116 and HeLa cells were treated with 15 µm of LW‐213 for 6 h. Cells were collected and crawled for immunofluorescence staining of cell nuclei for DAPI (blue) and LAMP1 protein (red). They were detected by confocal microscopy (FV1000; Olympus) with FV10‐ASW2.1 acquisition software (Olympus) at room temperature (original magnification × 1000; immersion objective × 100 × 40 with immersion oil type) (total cells in each group >100), ^*^ *p* < 0.05, ^**^ *p* < 0.01 compared to 0 µm LW‐213 group. G–I) The THP1 and HeLa cells were treated with 15 µm of LW‐213 for 6 h. Cells were collected and crawled for immunofluorescence staining of cell nuclei for DAPI (blue) and LAMP1 protein (green). They were detected by confocal microscopy (FV1000; Olympus) with FV10‐ASW2.1 acquisition software (Olympus) at room temperature (original magnification × 1000; immersion objective × 100 × 40 with immersion oil type) (total cells in each group >100), ns compared to 15 µm LW‐213 group. J) Flow cytometric analysis of Annexin V‐FITC/PI‐PerCP‐stained cell line of THP1 treated with 15 µm of LW‐213 with/without MβCD (0.05, 0.1, and 5 mm) for 9 h, ns compared to 15 µM LW‐213 group. K,L) The HeLa and shRAB7A or OE‐TBC1D15 HeLa cells were treated with 15 µm of LW‐213 for 6 h. Cells were collected and crawled for immunofluorescence staining of cell nuclei for DAPI (blue) and LAMP1 protein (red). They were detected by confocal microscopy (FV1000; Olympus) with FV10‐ASW2.1 acquisition software (Olympus) at room temperature (original magnification × 1000; immersion objective × 100 × 40 with immersion oil type) (total cells in each group >100). ^*^ *p* < 0.05, ^**^ *p* < 0.01, ns compared to 0 µm LW‐213 group, ^#^ *p* < 0.05, ^##^ *p* < 0.01 compared to Vector 0 µm LW‐213 group. (M) The THP1 cells were exposed to LW‐213 (15 µm) and CHX (50 µm) for 3, 6, and 9 h. (N) The THP1 cells were exposed to LW‐213 (15 µm) and BAF‐A1 (50 nm) or MG‐132(15 µm) for 9 h. O) The THP1 and MV4‐11 cells were treated with 15 µm of LW‐213 with BAF‐A1 (50 nm) for 6 h. Cells were collected and crawled for immunofluorescence staining of cell nuclei for DAPI (blue), LAMP1 protein (red) and SEC61α (green). THP1 cells were detected by confocal microscopy (FV1000; Olympus) with FV10‐ASW2.1 acquisition software (Olympus) at room temperature (original magnification × 1000; immersion objective × 100 × 40 with immersion oil type) (total cells in each group >100). MV4‐11 cells were detected by confocal microscopy (Leica Ultra‐High‐Resolution Laser Confocal) at room temperature (original magnification × 1000; immersion objective × 100 × 40 with immersion oil type). ^**^ *p* < 0.01, ns compared to 15 µm LW‐213 group. P) Flow cytometric analysis of Mag‐Fluo4‐AM‐stained cell line of THP1 treated with 15 µm of LW‐213 with/without BAF‐A1 (50 nm) for 1, 2, 3, 6, and 9 h. ^*^ *p* < 0.05, ^**^ *p* < 0.01 compared to 0 h LW‐213 group, ^#^ *p* < 0.05 compared to 9 h LW‐213 group. Q,R) Flow cytometric analysis of Annexin V‐FITC/PI‐PerCP‐stained cell line of Kasumi‐1, THP1 and MV4‐11 treated with 15 µm of LW‐213 with/without BAF‐A1 (50 nm) for 9 h. ^***^ *p* < 0.001 compared to 15 µm LW‐213 group. Data are shown as Mean ± S.E.M. from three independent experiments. ^*^ *p* < 0.05, ^**^ *p* < 0.01, ^***^ *p* < 0.001, ns indicates non‐significant.

Previous studies have demonstrated that the proximity between lysosomes and the ER is critical in facilitating Ca^2+^ exchange. Transcriptome sequencing Gene Ontology (GO) analysis of THP1 cells, following a 6 h treatment with LW‐213, showed differential gene function enrichment related to inner membrane‐linked organelles in both control and treated groups (Figure **4**A). Additionally, Gene Set Enrichment Analysis (GSEA) confirmed a significant enrichment of genes differentially expressed in lysosomes and the ER (Figure 4B). These RNA‐seq findings not only confirmed the primary organelles targeted by LW‐213 for its anti‐tumor effects but also provided insights for future research. The spatial arrangement of organelles is influenced by their intracellular transport mechanisms; given that the ER is diffusely distributed yet less mobile compared to the more flexible lysosomes, it was hypothesized that lysosome‐mediated transportation facilitates MCS formation with the ER. Bio‐transmission electron microscopy showed an increased perinuclear distribution of lysosomes (indicated by yellow arrows) in THP1 cells treated with LW‐213 (Figure 4C,D). Similarly, LW‐213 induced lysosome aggregation in HCT‐116 and HeLa cells with low TPC1 expression (Figure 4E,F). These results indicate that LW‐213 promotes lysosomal movement to the perinuclear region, facilitating Ca^2+^ exchange between organelles.

The perinuclear localization of lysosomes is influenced by two primary mechanisms: inhibition of anterograde transport and promotion of retrograde transport. Methyl‐β‐cyclodextrin (MβCD) has been reported to effectively alleviate the inhibition of anterograde transport,^[^ ^50^ ^]^ thereby facilitating the peripheral localization of lysosomes. To distribute more lysosomes toward the cell periphery, THP1 cells were pretreated with MβCD for 2 h prior to co‐treatment with LW‐213. Interestingly, no significant difference was observed in lysosome accumulation in the perinuclear region compared to the group treated only with LW‐213 (Figure 4G–I). Additionally, no notable difference was found in apoptosis between the co‐incubation group and the LW‐213 group (Figure 4J). These findings suggest that LW‐213 does not prevent lysosomal localization to the perinuclear region by inhibiting anterograde transport; rather, it enhances retrograde transport from peripheral regions to the nucleus. RAB7 and its negative regulator TBC1D15 are key proteins involved in this movement. To explore the role of LW‐213 in enhancing retrograde transport for lysosomal localization in the perinuclear region, HeLa cells with RAB7A knockdown and TBC1D15 overexpression were established (Figure S4A,B, Supporting Information). Immunofluorescence analysis showed that both knockdown and overexpression groups exhibited a more dispersed lysosomal distribution and a significant reduction in perinuclear density compared to the empty vector group (Figure 4K,L). Further investigations revealed that LW‐213 did not affect RAB7A expression but significantly shortened the half‐life of TBC1D15 in the presence of CHX (Figure 4M; Figure S4C–F, Supporting Information). Furthermore, to prevent the degradation of TBC1D15 protein, the use of the lysosomal degradation pathway inhibitor BAF‐A1 and the proteasome pathway inhibitor MG‐132 is recommended. LW‐213 has been demonstrated to promote the lysosomal degradation of TBC1D15 (Figure 4N). This reduction in TBC1D15 protein levels further diminishes its negative regulation on RAB7A, thereby enhancing retrograde transport of lysosomes.

Down‐regulation of TBC1D15 triggered a cascade of biological events, and the lysosomal degradation pathway inhibitor BAF‐A1 restored TBC1D15 expression. In theory, BAF‐A1 should prevent the downstream biological events triggered by LW‐213. As expected, immunofluorescence results showed that co‐incubation with BAF‐A1 and LW‐213 for 6 h in THP1 and MV4‐11 cells significantly reduced perinuclear aggregation of lysosomes (Figure 4O). Additionally, BAF‐A1 prevented perinuclear anchorage of lysosomes and decreased the likelihood of lysosome‐ER MCS formation, thus impeding calcium exchange between organelles and restoring ER calcium storage (Figure 4P), ultimately reversing apoptosis in the ER stress pathway (Figure 4Q,R). These findings indicate that enhanced retrograde transport of lysosomes initiates calcium exchange between lysosomes and the ER. Furthermore, TBC1D15 has been identified as a crucial regulator of lysosomal retrograde movement.

### 2.5 LW‐213 Binds LIMP2 to Downregulate TBC1D15 Expression Promoting Lysosomal Retrograde Transport

**Figure 5:**
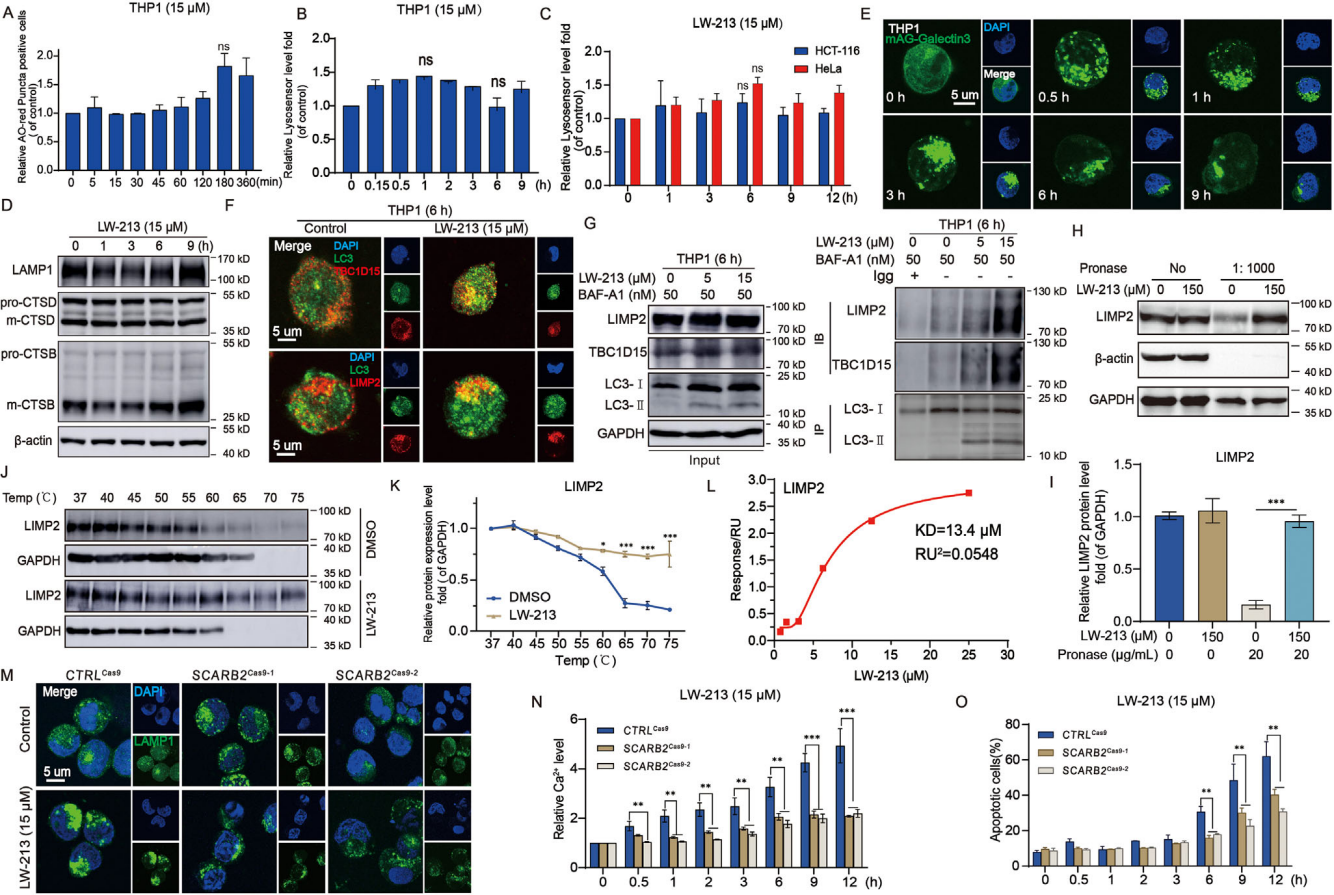
LW‐213 binds LIMP2 to downregulate TBC1D15 expression promoting lysosomal retrograde transport. A) Flow cytometric analysis of acridine orange‐stained cell line of THP1 treated with 15 µm of LW‐213 for 5, 15, 30, 45, 60, 120, 180, and 360 min. ns compared to 0 min group. B) Flow cytometric analysis of Lysosensor‐stained cell line of THP1 treated with 15 µm of LW‐213 for 0.15, 0.5, 1, 2, 3, 6, and 9 h. ns compared to 0 h group. C) Flow cytometric analysis of Lysosensor‐stained cell line of HCT‐116 and HeLa treated with 15 µm of LW‐213 for 1, 3, 6, 9, and 12 h. ns compared to 0 h group. D) The THP1 cells were exposed to LW‐213 (15 µm) for 1, 3, 6, and 9 h. E) THP1 cells transfected with mAG‐Galectin3 were treated with 15 µm of LW‐213 for 0.5, 1, 3, 6, and 9 h. Cells were collected and crawled for immunofluorescence. They were detected by confocal microscopy (FV1000; Olympus) with FV10‐ASW2.1 acquisition software (Olympus) at room temperature (original magnification × 1000; immersion objective × 100 × 40 with immersion oil type) (total cells in each group >100). F) THP1 cells were treated with 15 µm of LW‐213 for 6 h. Cells were collected and crawled for immunofluorescence staining of cell nuclei for DAPI (blue), TBC1D15 protein (red) and LC3 (green). They were detected by confocal microscopy (FV1000; Olympus) with FV10‐ASW2.1 acquisition software (Olympus) at room temperature (original magnification × 1000; immersion objective × 100 × 40 with immersion oil type) (total cells in each group >100). G) THP1 cells were cotreated with 5, 15 µm of LW‐213 and BAF‐A1(50 nm) for 6 h. H,I) Western Blot analysis of LIMP2 in THP1 treated by 150 µm LW‐213 and Pronase (20 mg mL^−1^, volume ratio 1:1000). β‐actin and GAPDH were used as a loading control. ^***^ *p* < 0.001 compared to 0 µm LW‐213 with Pronase (20 µg mL^−1^) group. J,K) CETSA melt curve of LIMP2 for heat treatment of THP1 cells in the absence and in the presence of LW‐213 (15 µm). GAPDH were used as a loading control. ^*^ *p* < 0.05, ^***^ *p* < 0.001 compared to DMSO group. L) The affinity of LIMP2 to LW‐213 at different concentrations was detected by SPR technique. M) Vector and LIMP2‐KO (#1, #2) THP1 cells were treated with 15 µm of LW‐213 for 6 h. Cells were collected and crawled for immunofluorescence staining of cell nuclei for DAPI (blue) and LAMP1 (green). They were detected by confocal microscopy (FV1000; Olympus) with FV10‐ASW2.1 acquisition software (Olympus) at room temperature (original magnification × 1000; immersion objective × 100 × 40 with immersion oil type) (total cells in each group >100). N) The Vector and LIMP2‐KO (#1, #2) THP1 cells were treated with 15 µm of LW‐213 for 0.5, 1, 2, 3, 6, 9, and 12 h, respectively. The Ca^2+^ indicator Fluo3‐AM measured cytoplasmic Ca^2+^ levels in each group of cells. ^**^ *p* < 0.01, ^***^ *p* < 0.001 compared to CTRL^Cas9^ group. O) Flow cytometric analysis of Annexin V‐FITC/PI‐PerCP‐stained cell line of Vector and LIMP2‐KO (#1, #2) THP1 cells were treated with 15 µm of LW‐213 for 0.5, 1, 2, 3, 6, 9, and 12 h. ^**^ *p* < 0.01 compared to CTRL^Cas9^ group. Data are shown as Mean ± S.E.M. from three independent experiments.^*^ *p* < 0.05, ^**^ *p* < 0.01, ^***^ *p* < 0.001, ns indicates non‐significant.

LW‐213 promotes the lysosomal degradation of TBC1D15, and ongoing research aims to define this specific mechanism. Our previous work (LC‐MS) identified lysosomes as the primary organelle interacting with LW‐213 (Figure S5E, Supporting Information). Given that enhanced lysosomal membrane permeability (LMP) can cause tumor cell death, we examined its effect on LMP by tracking the redistribution of acridine orange (AO) in THP1 cells for 6 h after LW‐213 treatment. Flow cytometry results showed no significant decrease in red fluorescent cells (Figure **5**A), indicating that lysosomal membranes remained intact. Further analysis using Lysosensor probes revealed minimal changes in lysosomal acidity (Figure 5B), with consistent results in HeLa and HCT‐116 cells (Figure 5C). We also assessed potential leakage and activity loss of cathepsins B and D. Western blots and immunofluorescence confirmed that LW‐213 did not alter the regulation or redistribution of active cathepsin B (CTSB) and cathepsin D (CTSD) from lysosomes to the cytoplasm (Figure 5D; Figure S5A,B, Supporting Information), indicating that apoptosis induced by LW‐213 does not depend on the LMP pathway.

The aggregation of Galectin 3 protein serves as an early indicator of lysosomal injury. Interestingly, significant Galectin 3 puncta formation was noted in THP1 cells transfected with mAG‐Galectin 3, within the first 3 h after LW‐213 treatment, and the puncta gradually diminished over time (Figure 5E). This suggests that LW‐213‐induced early lysosomal injury may be mitigated by a repair mechanism, ultimately preventing critical LMP. Consistent with prior research, TBC1D15 has been shown to mediate the anti‐tumor effects of LW‐213. Recent studies indicate that TBC1D15 supports membrane regeneration of damaged lysosomes, in conjunction with lysosomal membrane integration protein LIMP2 and autophagy core protein LC3B. Following LW‐213 exposure in THP1 cells, early lysosomal membrane damage was observed, leading to the recruitment of the LIMP2‐LC3B heterotrimer as a phagocytic receptor, which then recruited TBC1D15 to the damaged membrane (Figure 5F). Immunofluorescence results showed significant co‐localization between LC3B, TBC1D15, and LIMP2. To further verify the presence of the TBC1D15‐LC3B‐LIMP2 heterotrimer complex, immunoprecipitation experiments demonstrated an increased interaction between LIMP2‐LC3B‐TBC1D15 in THP1 cells treated with LW‐213 (Figure 5G). Importantly, persistent recruitment and attachment of TBC1D15 to the lysosomal membrane disrupted the critical balance needed for membrane repair and promoted excessive degradation within the lysosomal pathway.

The primary aim of this study was to identify the specific target protein responsible for the LW‐213‐induced polymerization of the LIMP2‐LC3B‐TBC1D15 heterotrimer. Given that LW‐213 primarily affects lysosomal homeostasis and considering potential drug targets, the results of LC‐MS protein identification showed that LIMP2 (SCARB2) may be a potential binding target for LW‐213 (Figure S5C,D, Supporting Information). Previous research indicated that although LW‐213 reduced TBC1D15 expression, it had no impact on LIMP2 expression (Figure S5G,H, Supporting Information). To further explore this, Drug Affinity Responsive Target Stability (DARTS) experiments were conducted, showing that LW‐213 did not alter Pronase sensitivity in TBC1D15 but protected LIMP2 from enzymatic hydrolysis by Pronase (Figure 5H,I; Figure S5F, Supporting Information). Additionally, results from the Cell Thermal Shift Assay (CTESA) demonstrated that LW‐213 preserved LIMP2 stability and reduced its degradation at high temperatures (Figure 5J,K). Furthermore, Surface Plasmon Resonance (SPR) analysis confirmed a binding affinity between LW‐213 and LIMP2 (Figure 5L; Figure S5I, Supporting Information).

The potential targeting of LIMP2 by LW‐213 was investigated through CRISPR/Cas9 knockout of the SCARB2 gene, which encodes LIMP2 (Figure S5J, Supporting Information), to assess its impact on the anti‐tumor effects of LW‐213. Initially, the knockout group exhibited significantly less apoptosis and lysosomal damage than the control group (Figure S5K,L, Supporting Information). Subsequently, peritubular localization of lysosomes was also significantly reduced (Figure 5M). As a result, Ca^2+^ exchange between the ER and lysosomes was impaired, leading to a reduction in cytoplasmic Ca^2+^ fluctuations (Figure 5N) and ultimately diminishing the apoptotic effect induced by LW‐213 (Figure 5O; Figure S5K, Supporting Information). In summary, LW‐213 targets LIMP2 to promote retrograde transport of lysosomes and down‐regulate TBC1D15 expression, facilitating peritubular aggregation of lysosomes and providing a structural basis for subsequent organelle interactions.

### 2.6 Validation of the Anti‐Tumor Efficacy of LW‐213 by Targeting LIMP2 In Vivo

**Figure 6:**
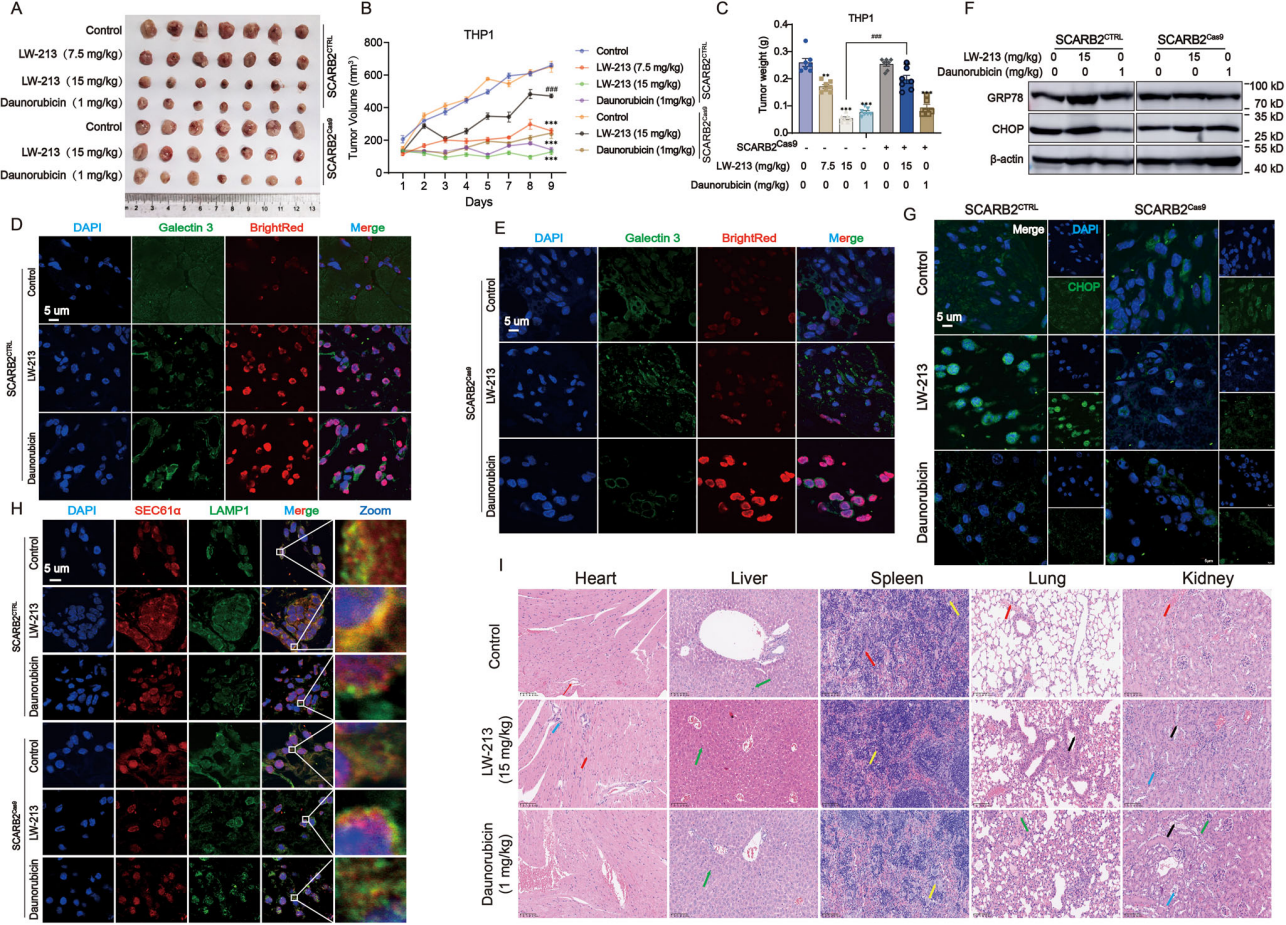
Validation of the anti‐tumor efficacy of LW‐213 by targeting LIMP2 in vivo. A,B) Tumor volume was measured every day and quantified as 0.5 × length × width × width. The volume was expressed as mean ± SD (n = 7) and represented as tumor volume–time curves to show. ^***^ *p* < 0.001 compared to Control group, ^###^ *p* < 0.001 compared to SCARB2^CTRL^ LW‐213 (15 mg kg^−1^) group. C) After 10 days of administration, mice were sacrificed, and tumors were weighted. The representative tumors were shown. Tumor weight in each group was expressed as mean ± SD (n = 7). ^**^ *p* < 0.01, ^***^ *p* < 0.001 compared to SCARB2^CTRL^ LW‐213 (0 mg kg^−1^) group, ^###^ *p* < 0.001 compared to SCARB2^CTRL^ LW‐213 (15 mg kg^−1^) group. D,E) Tumor tissue sections were collected and crawled for immunofluorescence staining of cell nuclei for DAPI (blue), Galectin 3 (green) and BrightRed (red). They were detected by confocal microscopy (FV1000; Olympus) with FV10‐ASW2.1 acquisition software (Olympus) at room temperature (original magnification × 1000; immersion objective × 100 × 40 with immersion oil type) (total cells in each group >100). F) The expression levels of GRP78 and CHOP were analyzed in tumor tissue protein by western blot. β‐actin was used as a loading control. G) Tumor tissue sections were collected and crawled for immunofluorescence staining of cell nuclei for DAPI (blue) and CHOP (green). They were detected by confocal microscopy (FV1000; Olympus) with FV10‐ASW2.1 acquisition software (Olympus) at room temperature (original magnification × 1000; immersion objective × 100 × 40 with immersion oil type) (total cells in each group >100). H) Tumor tissue sections were collected and crawled for immunofluorescence staining of cell nuclei for DAPI (blue), SEC61α (red) and LAMP1 (green). They were detected by confocal microscopy (FV1000; Olympus) with FV10‐ASW2.1 acquisition software (Olympus) at room temperature (original magnification × 1000; immersion objective × 100 × 40 with immersion oil type) (total cells in each group >100). I) H&E staining for toxicity detection of *BALB/c* nude mice organs. Data are shown as Mean ± S.E.M. from three independent experiments. ^*^ *p* < 0.05, ^**^ *p* < 0.01, ^***^ *p* < 0.001, ns indicates non‐significant.

The initial in vitro studies revealed the molecular mechanisms of LW‐213. Subsequently, the pharmacodynamics and safety profile of LW‐213 were assessed using cell‐derived xenografts in *BALB/c* nude mice inoculated with wild‐type and LIMP2‐knockout THP1 cell lines. Blood routine tests were conducted on *ICR* mice (Figure S6A, Supporting Information). The pharmacodynamics results showed that both 7.5 and 15 mg kg^−1^ of LW‐213 significantly inhibited tumorigenesis in wild‐type THP1 cells. However, no significant difference was noted between the Daunorubicin group and the wild‐type group in their inhibitory effects in the LIMP2‐knockout THP1 animal model; whereas, the 15 mg kg^−1^ of LW‐213 showed a considerably reduced pharmacodynamic response in LIMP2‐knockout group (Figure **6**A–C). The anti‐AML effect of LW‐213 were consistent both in vitro and in vivo. Final mouse weights were significantly lower than initial weights only in those treated with Daunorubicin, according to weight‐time curves during administration for both animal models; however, no inhibitory effect on mouse weight was observed in the LW‐213 treatment group (Figure S6B, Supporting Information).

The anti‐leukemia mechanism of LW‐213 was investigated at a molecular level through a TUNEL Bright Red staining experiment, which assessed apoptosis in tumor tissue sections. Immunofluorescence results indicated that both 15 mg kg^−1^ of LW‐213 and 1 mg kg^−1^ of Daunorubicin induced apoptosis in THP1 cell‐derived tumors without causing Galectin 3 aggregation. In a solid tumor model with LIMP2‐knockout THP1 cells, LW‐213′s apoptotic effect significantly decreased at the same dose but remained consistent for Daunorubicin‐treated cells (Figure 6D,E). These findings suggest that LW‐213‐induced apoptosis is independent of lysosomal damage and that LIMP2 knockout reduces the tumor cell response to its apoptotic effect.

To establish the link between tumor cell apoptosis and ER stress, Western Blot analysis showed a marked increase in GRP78 and CHOP expression in LW‐213‐treated tumor tissues compared to controls in wild‐type LIMP2 animal models. However, this upregulation by LW‐213 was diminished in LIMP2 knockout models, with no similar trend observed for Daunorubicin (Figure 6F; Figure S6C,D, Supporting Information). Fluorescence labeling of CHOP in tumor cells was then used to examine intracellular distribution differences among treatment groups. Immunofluorescence results demonstrated that nuclear translocation of CHOP was more pronounced in the LW‐213 group compared to both vehicle and Daunorubicin groups; however, LW‐213‐induced intranuclear localization of CHOP was inhibited in mouse models using LIMP2‐knockout cells (Figure 6G; Figure S6E, Supporting Information). These experimental findings collectively indicated that LW‐213′s apoptotic effect on tumor cells was linked to the activation of ER stress.

The impact of LW‐213 on the spatial arrangement of lysosomes and ER was explored in vivo. Immunofluorescence results showed that the LW‐213 group prompted co‐localization between lysosomes and ER in tumor cells within wild‐type THP1‐inoculated solid tumors, aligning with in vitro findings. In contrast, the Daunorubicin group did not exhibit this phenomenon. In LIMP2‐knockout THP1‐inoculated solid tumors, the LW‐213 group's ability to induce co‐localization between these two organelles was significantly reduced (Figure 6H; Figure S6F, Supporting Information). This suggested a potential interaction between lysosomes and ER during LW‐213′s anti‐AML effects.

The in vivo safety evaluation of LW‐213 primarily focused on assessing organ injury through H&E staining and blood routine analysis to detect changes in blood cell count and composition. Pathological tissue examination revealed that, compared to the vehicle group, LW‐213 exhibited no significant organ‐targeted toxicity (Figure 6I). Furthermore, analysis of blood routine data indicated that doses of 7.5 and 15 mg kg^−1^ of LW‐213 had a lesser impact on blood cells than Daunorubicin (Figure S6G, Supporting Information). Overall, these initial findings have demonstrated that LW‐213 exhibits potent anti‐AML effects in vivo with reduced toxicity compared to Daunorubicin.

## 3 Discussion

With advancements in molecular biology technology, a growing number of intercellular communication pathways have been identified. This study has demonstrated that lysosomes can modify their transport modes to interact with the ER. It has also shown that Ca^2+^ signals from lysosomal TPC1 can initiate the release of IP3R1 Ca^2+^ in the ER, leading to lethal Ca^2+^ fluctuations through a cascade amplification process. The flavonoid LW‐213 has proven its ability to trigger these biological events in tumor cells, displaying significant anti‐tumor efficacy. By targeting the lysosomal membrane integrin LIMP2, LW‐213 enhanced the recruitment of TBC1D15 to lysosomes, which promoted degradation via the lysosomal pathway and facilitated retrograde transport of lysosomes. Consequently, it extended the formation of MCSs between lysosomes and the ER, significantly improving the efficiency of Ca^2+^ exchange between these organelles. Furthermore, we have verified that this anti‐tumor approach is selective and especially effective in tumors with high levels of TPCN1 expression.

In recent years, extensive research has explored Ca^2+^ communication between organelles. These studies indicate that interactions between Ca^2+^ signals from the ER and mitochondria can influence mitochondrial structure and energy metabolism.^[^ ^51^, ^52^ ^]^ The proximity of the ER to the plasma membrane enhances its capacity for additional Ca^2+^ storage. Ca^2+^‐mediated fusion and division of endosomes initiate the exchange of calcium signals between endosome/lysosomes and the ER. MCSs between late endosome/lysosomes and the ER provide a structural basis for maintaining an “acidic calcium pool” within lysosomes.^[^ ^53^, ^54^ ^]^ These insights have laid a solid theoretical foundation for developing anti‐tumor strategies and identifying drug targets. However, due to complex inter‐organelle connections, their impact on cell fate has become more complex, affecting more than just individual organelle function or structure. Even with optimal physiological interactions facilitated by small‐molecule compounds or genetic manipulation, deviations in signal transmission can occur. Therefore, it is vital to explore how to regulate dynamic organelle connections to ensure accurate and efficient information exchange.

Although tumor‐specific agents targeting lysosome‐ER calcium signaling crosstalk remain unexplored at this stage, modulating organelle interactions has emerged as a critical anticancer strategy. Current research advances primarily focus on identifying key targets^[^ ^55^, ^56^ ^]^(e.g., TRPML1‐mediated calcium homeostasis regulation, cholesterol transport‐driven mitochondrial reprogramming), developing cascade‐damage drug delivery systems^[^ ^57^ ^]^ (e.g., photo‐switchable nanoparticles LD‐UCNP@CS‐Rb‐TPP that induce dual‐organelle damage), and designing dynamic monitoring probes^[^ ^58^ ^]^ (e.g., dual‐organelle targeting probes). These advances not only establish organelle interactions as a significant direction for novel drug development but also provide theoretical and technical support for the advancement of targeted intervention agents. This study pioneers the characterization of the natural compound LW‐213 as a specific modulator of lysosome‐ER calcium signaling crosstalk, not only directly validating the feasibility of targeting organelle interactions as a therapeutic strategy but also establishing a mechanism‐guided theoretical framework to underpin innovative drug development.

Lysosomes have become a significant target for anti‐tumor therapies, leading to the development of numerous drugs that leverage lysosomal function. The efficient operation of lysosomes is essential for rapidly dividing tumor cells that rely heavily on them. During tumor progression, substantial changes occur in the lysosomal compartment, including variations in quantity and size, repositioning from the perinuclear area to the peripheral region, and enhanced enzyme expression or activation. Tumor cell death may be induced through lysosome‐mediated mechanisms by altering membrane permeability (Lysosomal‐Mediated Cell Death or LCD).^[^ ^59^, ^60^ ^]^ However, no anti‐tumor strategies that modify cytoplasmic localization of lysosomes have been identified to date. Our study reveals that LW‐213 primarily causes tumor cell death by enhancing perinuclear lysosomal distribution. Unlike other methods affecting cytoplasmic localization, such as the reversible glutamine depletion‐induced starvation, LW‐213‐induced aggregation near the nucleus is irreversible and permanent (Figure S4C,D, Supporting Information). This feature prolongs Ca^2+^ exchange between the ER and lysosomes, increasing stability within their “calcium microdomain.” This might help explain why replicating LW‐213′s full effect using cell starvation alone is unfeasible.

Moreover, mTOR complex inhibitors, such as Rapamycin and Torin1, also promote peritubular lysosomal distribution;^[^ ^61^, ^62^ ^]^ however, they do not replicate the apoptotic effect seen with LW‐213 (Figure S4E, Supporting Information). When amino acid signals to lysosomes are disrupted by mTOR inhibitors, cell survival becomes dependent on macro‐autophagy mediated by transcription factor EB (TFEB).^[^ ^63^ ^]^ Nevertheless, LW‐213 treatment does not affect the subcellular location of the mTOR complex relative to lysosomal distribution; specifically, it prevents co‐localization between mTOR and lysosomes (Figure S4F, Supporting Information). Therefore, under LW‐213 stimulation, mTOR does not respond appropriately to stress, leading to a TFEB‐independent cellular rescue pathway, facilitated by LIMP2 recruiting TBC1D15 for lysosomal regeneration. Although perinuclear lysosome localization seems consistent, their feedback mechanisms vary significantly and are critical in determining cellular fate. These differences encourage further research into precise lysosomal localization regulation for improved anti‐tumor effects.

In conclusion, this study demonstrates Ca^2+^ signal interaction at lysosomal‐ER MCSs and discloses the dynamic regulatory role of TPC1 on IP3R1 for the first time. It also introduces a novel anti‐tumor approach where lysosomal TPC1 Ca^2+^ signaling reduces ER Ca^2+^ storage and leads to cell death through the ER stress pathway. As a molecular probe, LW‐213 binds directly to its target, LIMP2, in AML‐dominant tumor cells. It facilitates Ca^2+^ exchange between lysosomes and the ER by promoting the formation of a LIMP2‐LC3B‐TBC1D15 trimer. By exploring LW‐213′s mechanism, we have confirmed the crucial role of organelle interaction in cellular demise, presenting a novel target and potential drug candidate.

## 4 Experimental Section

### Compounds and Reagents

LW‐213 was synthesized from Chrysin (Shanxi Ciyuan Biotech Co., Ltd, China). The final compound was a yellow solid powder at purity >99.5%. For in vitro experiments, LW‐213 was dissolved in dimethyl sulfoxide (DMSO, Sigma‐Aldrich, St. Louis, MO, USA) to the concentration of 0.02 M, stored at ‐80 °C and diluted to a suitable concentration with RPMI‐1640 medium (GIBCO, Carlsbad, CA, USA). Cells treated with the highest concentration of DMSO was as control in corresponding experiments.

For in vivo experiments, LW‐213 was prepared as intraperitoneal injection administration formulation by Dr. Xue Ke from College of Pharmacy, China Pharmaceutical University. 2‐Aminoethyl diphenylborinate (2‐APB) (HY‐W009724), CDN‐1163 (HY‐101455), BAPTA‐AM (HY‐100545), Ned‐19 (HY‐103316A), BAF‐A1 (HY‐100558), MβCD (HY‐101461), MG‐132 (HY‐13259 ), Cycloheximide (HY‐12320), Rapamycin (HY‐10219), Torin1 (HY‐13003) were purchased from MedChemExpress.

### Cell Culture

Cell lines Kasumi‐1, MV4‐11, THP1, SKNO‐1, Hut‐102, BXPC3, LEWIS, HEK293T, HeLa, HCT‐116, HT‐29, HepG2, and MCF‐7 cells were purchased from Cell Bank of Shanghai Institute of Biochemistry & Cell Biology. All the cell lines were authenticated under short tandem repeat analysis by the suppliers. Then the cells were expanded and stored in liquid nitrogen upon receipt, and each aliquot was passaged for fewer than 25–30 times in laboratory. Kasumi‐1, MV4‐11, THP1, SKNO‐1, Hut‐102, HCT‐116, HT‐29, and HeLa Cells were cultured in RPMI‐1640 medium, HEK293T, HepG2, BXPC3, LEWIS, and MCF‐7 cells were cultured in DMEM medium, supplemented with 10% heat‐inactivated FBS (Thermo Fisher Scientific, Waltham, MA), 100 U mL^−1^ of benzyl penicillin, and 100 U mL^−1^ of streptomycin in a humidified environment (Thermo Fisher Scientific, Waltham, MA) with 5% CO_2_ at 37 °C.

### AML Primary Cell Treatment

After anticoagulation treatment of AML whole blood cells derived from clinical sources, they were transported at low temperature to the super‐clean bench for subsequent operations. Take a sufficient amount of 5 mL of fresh blood and place it in a 15 mL sterilized centrifuge tube, and add sterile PBS to dilute the blood at a ratio of 1:1. Take 5 mL of lymphocytic separation fluid and place it in a pre‐cooled 15 mL sterilized centrifuge tube for later use. Aspirate the diluted blood and slowly add it along the tube wall 1 cm above the separation fluid, enabling the diluted blood to overlap the separation fluid and form a distinct interface. At room temperature, centrifuge at 1500 rpm for 20 min. At this point, a lymphocyte layer in the form of a white membrane exists between the plasma layer and the lymphocytic separation fluid. Carefully aspirate the intermediate white membrane‐like mononuclear cell layer, attempting to aspirate all the mononuclear cells. Wash twice with sterile PBS, centrifuge at 1500 rpm for 10 min, remove the supernatant, add 1640 complete medium to resuspend the cells, and incubate them at 37 °C and 5% CO_2_ until ready for use.

### CCK8 Assay

Cells (5000 cells per well) were seeded in 96‐well plates, and then treated with LW‐213 at various concentrations for 48 h. Then, 20 µL CCK8 solution was added to each well and incubated for 4 h. Cells treated with equivalent amounts of DMSO (0.1%) were used as a negative control. The absorbance was read at 450 nm with a SynergyTM HT multimode reader (BioTek, Vermont, USA). The average value of the optical density of five wells was used to determine cell viability by the following formula:

(1) $$\mathrm{Survival}\;\mathrm{rate}\;(\%)=OD_{\left(\mathrm{treatment}\;\mathrm{group}\right)}\;/OD_{\left(\mathrm{control}\;\mathrm{group}\right)}\;\times \;100\%$$

The IC_50_ value was defined as the concentration that caused a 50% inhibition in cell viability and was calculated by the logit method.

### Flow Cytometric Analysis of Apoptosis

Cells were collected and processed with Annexin V/PI Cell Apoptosis Detection Kit (Vazyme Biotec, Nanjing, China) for 10 min at room temperature in the dark, according to the protocols. Data acquisition and analysis were performed by Becton‐Dickinson FACS Calibur flow cytometry and CellQuest software. The cells stained by neither Annexin V nor PI were regarded as survival.

### Detection of Intracellular Calcium Level

Cells were collected with 1 µm Fluo‐3AM (S1056; Beyotime Institute of Biotechnology, Nanjing, China), bound to Ca^2+^, generating intense fluorescence. Cells were resuspended with 500 µL PBS after 30 min of incubation in 37 °C darkness according to the manufacturer's protocol, and the fluorescence intensity of cells was determined using FACS Calibur Flow cytometry (Becton Dickinson, San Jose, CA. 488/525 nm).

### Plasmid Transfection and RNA Interference

TPCN1, RAB7A shRNA, and negative control (NC) constructs used pLV3ltr‐puroU6 vector system were obtained from Corues Biotechnology (Nanjing, China) for RNA interference. HEK293T cells were transfected with TPCN1 or RAB7A shRNA constructs and NC along with a lentiviral cocktail and HG transgene reagents for 48 h following the manufacturer's instructions for the lentiviral packaging kit (YEASEN Biotech). Afterward, the viral supernatant was collected. Then, the viral supernatant infects the target cells. Add 2 µg mL^−1^ puromycin to select shRNA to construct cells.

For transfection plasmid, RA‐SEC61β lentiviral vector plasmid was purchased from MIAOLING BIOLOGY (Wuhan, China). mAG‐Galectin3 plasmid was obtained from Addgene (Cambridge, MA, USA). Lentiviral vector plasmid was transfected into HEK293T cells for 48 h. Viral supernatant was then collected. The viral supernatant was used to infect cells. To select for the cells that were stably expressing constructs, cells were incubated in RPMI‐1640 medium with 10 % FBS and 2 µg mL^−1^ of puromycin for 48 h after lentivirus infection. After selection cells, stably infected pooled clones were harvested for use.

### Crispr/Cas9 Genome Editing

As mentioned, SCARB2‐knockout cells were constructed by using CRISPR/Cas9 system. In summary, the pLenti CRISPR/Cas9 V2 vector expressing sgRNA targeting SCARB2 was transfected into 293T cells, viral fluid was to infect THP1 cells, and SCARB2‐knockout cells were screened with puromycin. Western blotting confirmed successful knockout of LIMP2. The sequences of sgRNAs were as followed, sgRNA1: GGGGTGAAGAAGGTGGGCTC; sgRNA2: TCACTGATACTCTCACACGT; sgRNA3: CGGCCGAAGACATATGGACT, which were designed and synthesized by MIAOLING BIOLOGY (Wuhan, China).

### Immunofluorescence Analysis

Cells were collected, rinsed twice in PBS and blotted onto coverslips. Cells were then fixed in ice‐cold methanol for 15 min and permeabilized with 0.2% Triton X‐100 (X100‐500 ML, Sigma‐Aldrich) for 30 min. 3% bovine serum albumin (A3858‐100G, Sigma‐Aldrich) was blocked for 1 h at room temperature and incubated with primary anti‐Calreticulin antibody (1:200) overnight at 4 °C. The samples were washed three times and followed by co‐incubation with Alexa Fluor 488 Goat anti‐Rabbit IgG (H+L) antibody (1:500) or Alexa Fluor 546 Goat anti‐Rabbit IgG (H+L) antibody (1:500) for 1 h at 37 °C. Then slides were washed and counterstained with DAPI (Beyotime, China) working solution (100 µg ml^−1^) for 20 min at room temperature. The images were captured with a confocal microscope at 1000 × magnification (Leica TCS STED; Berlin, Germany. FV1000; Olympus, Tokyo, Japan).

### Endoplasmic Reticulum Calcium Detection

The cells were collected in vitro, and the endoplasmic reticulum cavity free calcium ion detection probe was loaded according to the protocol of the Mag‐Fluo4 AM kit (AAT‐B20401, AAT Bioquest, USA). Calcium ion changes within the endoplasmic reticulum were determined based on the fluorescence intensity of the probe (Ex:490; Em:525; Cutoff:515) after the operation was completed, and the assay included fluorescence microscopy or the FITC channel of a flow cytometry to obtain the fluorescence intensity changes.

### Western Blot Analysis

Cells were collected and washed 3 times with PBS. Lysed cells were lysed with RIPA buffer (Thermo scientific, USA) and protease inhibitors (PMSF, NaF, leupeptin, and dithiothreitol) for 45 min on ice. Lysed cells should be shaken every 25 min. The cells were then placed through 13,000 rpm (5430R; Eppendorf, Hamburg, Germany) for 20 min at 4 °C. The protein concentration in the supernatant was quantified using a BCA kit and a Varioskan multimode microplate spectrophotometer (Thermo, Waltham, MA). Equal amounts of protein lysate products (50 µg) were subjected to sodium dodecyl sulfate‐polyacrylamide gel electrophoresis (SDS‐PAGE) system at 120 V for 1.5 h. The proteins were then transferred to nitrocellulose filter membranes (NC) (Millipore, Boston, MA, USA). Blots were closed with 3% BSA in PBS for 1 h at room temperature and incubated overnight at 4 °C with an antibody specific for the indicated primary antibody. The blots were washed 3 times with PBST and probed with IRDyeTM 800‐labeled secondary antibody probes at 37 °C for 1 h. The blots were reacted with enhanced chemiluminescence (ECL) reagent (Bio‐Rad, Hercules, CA, USA) and signal was detected with Amersham Imager 600 (GE, Piscataway, NJ, USA).

Primary antibodies for TPC1 (23758‐1‐AP, RRID:AB_2879317), RAB7 (55469‐1‐AP, RRID:AB_11182831), ARL8B (13049‐1‐AP, RRID: AB_2^059000), TBC1D15 (17252‐1‐AP, RRID:AB_2878370), LC3 (14600‐1‐AP, RRID: AB_2137737), LIMP2 (27102‐1‐AP, RRID: AB_2880756), LAMP1 (67300‐1‐Ig, RRID:AB_2882564), SEC61α (24935‐1‐AP, RRID: AB_2879807), STIM1 (11565‐1‐AP, RRID: AB_2302808), ORAI1 (66223‐1‐Ig, RRID: AB_2881614), mTOR (66888‐1‐Ig, RRID: AB_2882219), CTSB (12216‐1‐AP, RRID: AB_2086929), SERCA2 (67248‐1‐Ig, RRID: AB_2882525), SERCA3 (13619‐1‐AP, RRID: AB_2061448), GRP78 (11587‐1‐AP, RRID: AB_2119855), LAMP2 (66301‐1‐Ig, RRID: AB_2881684), β‐Actin (66009‐1‐Ig, RRID: AB_2687938), GAPDH ( 60004‐1‐Ig, RRID: AB_2107436), CTSD (21327‐1‐AP, RRID: AB_10733646), CHOP (15204‐1‐AP, RRID: AB_2292610) were obtained from Proteintech Technology (Wuhan, China). IRDye 800‐conjugated secondary antibodies were purchased from Rockland (Philadelphia, PA, USA).

### RNA Sequencing

THP1 cells were treated with LW‐213 (15 µm) and without LW‐213 for 6 h. THP1 cells were collected separately, and the appropriate amount of RNA extraction reagent Trizol (Vazyme, Nanjing, China) was added, and the subsequent sequencing and analysis were performed by GENE DENOVO (Guangzhou, China), and the relevant analysis reports were stored in the database of the Omicshare cloud platform under the project number GHRL22050395.

### Transmission Electron Microscopy (TEM) Imaging

THP1 cells were planted in dishes and given DMSO or LW‐213 for the duration of the experiment. After collected and washed, 2.5% glutaraldehyde was added to 0.1 µm phosphate buffer and the cells was fixed overnight at 4 °C and incubated with osmium tetraoxide for 2 h at 4 °C. Samples were dehydrated with upgraded ethanol (30%–95%), then epoxy resin was used to embed it (Solarbio Life Sciences, G8590). The ultrathin slices were then stained with lead citrate and 3% aqueous uranyl acetate. The cells were examined using a transmission electron microscope (Hitachi H‐7000FA, Tokyo, Japan).

### Detection of Lysosensor/Lysotracker Level

Cells were labelled with LysoTracker Red (KeyGen Biotech) or Lysosensor DND‐189 (YEASEN Biotech) for 20 min at 37 °C. Cells were then washed and fluorescence was detected under a flow cytometry.

### Acridine Orange (AO) Staining

The cells were stained with AO before collecting and incubated at 37 °C for 20 min. The fluorescence was detected under a laser confocal scanning microscope or flow cytometry. AO produces red fluorescence (E_m_:650 nm) in lysosome, and green fluorescence (E_m_:530 nm) in cytoplasm and nuclear.

### Co‐Immunoprecipitation Analysis

Cells were pretreated with LW‐213 (5 µm/15 µm) or without LW‐213 in the presence of BAF‐A1. Cell lysates were incubated overnight at 4 °C with appropriate concentrations of antibodies and 20 µL protein A/G – conjugated beads (HY‐K0202; MedChemExpress). After washing three times with RIPA buffer (Thermo Fisher), samples were collected and resuspended in 20 µL 2 × loading buffer and boiled for 10 min. Then western blot analysis was performed.

### Cellular Thermal Shift Assay (CETSA)

Briefly, THP1 cells were cultured with LW‐213 (15 µm) and DMSO for 6 h, respectively. The mixture was then divided into 100 µL equal test tubes and heated according to the established procedure. The cells were lysed in a thermal cycler, then frozen several times with liquid nitrogen and centrifuged for 20–25 min. After centrifugation, 5 × loading buffer was added and boiled for Western blot analysis.

### Drug Affinity Responsive Target Stability (DARTS)

The DARTS was performed using the same method as in previous experiments with slight variations. THP1 cells were removed and lysed with M‐PER (Thermo Fisher Scientific). After centrifugation, 1 × TNC buffer (50 mM Tris, 140 mM NaCl, 10 mM CaCl_2_) was added to the cell lysate. The M‐PER lysate was used to dilute the samples and was divided into experimental and control groups. The experimental group was treated with 150 µm LW‐213 and the control group was treated with an equal volume of DMSO and rotated overnight at 4 °C. Lysates were digested with 1:1000 pronase (20 mM; Sigma‐Aldrich, Taufkirchen, Germany) at 37 °C for 30 min, followed by the addition of protease inhibitors. Sampling buffer was then added and the proteins were denatured in a boiling water bath. Subsequent western blot experiments were used to detect changes in target proteins.

### Surface Plasmon Resonance (SPR) Binding Assay

SPR binding studies were performed on a Biacore S200 instrument (Cytiva; USA) according to the method described by official website. Briefly, full‐length recombinant human LIMP2 proteins were immobilized on CM5 sensor chips (GE Healthcare, Piscataway, NJ) by standard amine coupling, respectively. Serial concentrations of LW‐213 were prepared. The binding of different concentrations of LW‐213 to LIMP2 was detected, and then the kinetic binding curves were plotted to calculate the binding constant of the drug to the target proteins. Data were analyzed using Biacore S200 Evaluation software 1.1.1. Recombinant human LIMP2 protein (RP00086) was purchased from ABclonal (Wuhan, China). Recombinant Human LIMP II/SCARB2/CD36L2 Protein was produced by HEK293 expression system. The target protein was expressed with sequence (Arg27‐Thr432) of human LIMPII/SR‐B2 (Accession #NP_0 05497.1) fused with an Fc, 6×His tag at the C‐terminus.

### Quantitative Real‐Time Polymerase Chain Reaction (qRT‐PCR)

qRT‐PCR was performed according to the manufacturer's instructions. The primer sequences were as follows:

TPC1.
Forward 5′‐GGGAGATGAATTACCAAGAGGC‐3′
Reverse 5′‐GTGGCGTGGACATAGATGCC‐3′
TPC2.
Forward 5′‐TACCGCAGCATCCAAGTCG‐3′
Reverse 5′‐CACCCGGTGGTTGATGGAG‐3′
TRPML1.
Forward 5′‐GCGCCTATGACACCATCAA‐3′
Reverse 5′‐TATCCTGGACTGCTCGAT‐3′
SERCA2.
Forward 5′‐ATGGGGCTCCAACGAGTTAC‐3′
Reverse 5′‐TTTCCTGCCATACACCCACAA‐3′
SERCA2a.
Forward 5′‐GAGAACGCTCACACAAAGACC‐3′
Reverse 5′‐ACTGCTCAATCACAAGTTCCAG‐3′
SERCA2b.
Forward 5′‐TCGTTTTGGCTTGGTTCGAG‐3′
Reverse 5′‐CACCCACGATTGCATTGGC‐3′
SERCA3.
Forward 5′‐CTGGTCATCATGCTGATCCTC‐3′
Reverse 5′‐CAGCGTGGTGGACTTGATCT‐3′
GAPDH.
Forward 5′‐GCAGGGGGGAGCCAAAAGGG‐3′
Reverse 5′‐TGCCAGCCCCAGCGTCAAAG‐3′

### Live Cell Imaging System Imaging

HeLa cells were loaded with ER‐tracker and Lyso‐tracker live cell fluorescent probes and incubated for 30 min in a 5% CO_2_ cell incubator at 37 °C, away from light. After loading the probe, the staining solution was removed by centrifuge, and the appropriate amount of THP1 cells were suspended in 200 µL fresh culture solution containing LW‐213 (15 µm), inoculated into confocal laser glass culture dish, and immediately placed into the cell culture module of living cell workstation. The dynamic interaction between lysosome and endoplasmic reticulum was observed by means of live cell microscopy (Andor BC43, Oxford Instrument, Abingdon, UK).

### Xenograft Tumor Mouse Model

*BALB/c* nude mice aged 4 to 6 weeks (Slaccas Shanghai Laboratory Animal, Shanghai, China) were inoculated subcutaneously with wild‐type or LMP2‐knockout THP1 cells (5 × 10^5^). Animals were assigned to experimental groups using simple randomization. Responses were then scored by an experimenter blinded to injection condition and experimental cohort. LW‐213 was injected subcutaneously once daily at 7.5 and 15 mg kg^−1^; Daunorubicin was administered via tail vein injection twice weekly at 1 mg kg^−1^. Mice were observed daily for tumor volume changes during treatment. After nine days, they were euthanized by cervical dislocation, and their tumors and organs were collected for further studies.

### Blood Routine Test

Female ICR mice aged 6 to 8 weeks (Slaccas Shanghai Laboratory Animal, Shanghai, China) were divided into four groups: saline group, LW‐213 (7.5 mg kg^−1^) group, LW‐213 (15 mg kg^−1^) group and Daunorubicin (1 mg kg^−1^) group, with 4 mice in each group. The saline group and LW‐213 group were given intraperitoneal injection once a day, and the Daunorubicin group was given caudal venous administration once every two days. After 2.5 weeks of continuous administration, 20 µL blood was taken from the cheeks of mice and injected into the heparin anticoagulation tube. PE‐6800VET automatic blood analyzer (PROKAN, ShenZhen, China) was used for detection and analysis.

### TUNEL BrightRed Cell Apoptosis Detection

The tumor tissue sections were dewaxed at 65 °C, soaked in xylene for 10 min, and hydrated with gradient alcohol. The same procedure as immunofluorescence was followed until the end of closure, and immersed for 3 × 5 min in PBS. Equilibration with 100 µL 1 × Equilibration Buffer at room temperature for 20 min. The equilibrium solution was removed, and 50 µL TdT working solution was added to each climbing tablet to completely cover the sample area, and incubated at 37 °C in a wet box for 60 min, and washed three times with PBS for 5 min each time. The subsequent co‐staining process with antibody was the same as immunofluorescence method.

### Statistical Analysis

All results were expressed as mean ± standard error of the mean (S.E.M). The data shown were obtained from triplicate independent parallel experiments. Statistical analyses of multiple‐group comparisons were performed by one‐way analysis of variance (ANOVA) followed by the Bonferroni post‐test. Comparisons between two groups were analyzed using two‐tailed Student *t* tests. The significance of differences was indicated as *P < 0.05, **P < 0.01, and ***P < 0.001.

### Ethics Approval and Consent to Participate

All experiments using animal samples were approved by Institutional Animal Care and Use Committee of Nanjing Medical University. Ethics number: IACUC‐2103045. Human samples were approved by the institutional research ethics committee of The First Affiliated Hospital of Nanjing Medical University. Ethics number: 2023‐SR‐284.

## Conflict of Interest

The authors declare no conflict of interest.

## Author Contributions

M.‐Y.Z. and Y.‐J.G. contributed equally to this work. M.‐Y.Z. performed conceptualization and wrote, reviewed, and edited the draft. Y.‐J.G. performed data curation. Y.‐Q.Z. performed investigation. H.‐Z.W. performed methodology and formal analysis. H.‐D.W. performed supervision. H.‐Y.C. performed formal analysis. Y.‐X.J. performed validation. H.L. and H.H. performed project administration.

## Supporting information



Supporting Information

Supplemental Video 1

Supplemental Video 2

Supplemental Video 3

Supplemental Video 4

Supplemental Table

Supplemental Data

Supplemental Data

## Data Availability

All data included in this study are available upon request by contact with the corresponding author.
