# Supplementary material for: Activation of Lysosomal Retrograde Transport Triggers TPC1‐IP3R1 Ca2+ Crosstalk at Lysosome‐ER MCSs Leading to Lethal Depleting of ER Calcium
Source: Adv Sci (Weinh). 2025 Jul 25;12(39):e15313. doi: 10.1002/advs.202415313 (PMC12533322; doi:10.1002/advs.202415313)
Supplement: Supplementary file 1 — Supporting Information [file ADVS-12-e15313-s005.docx]

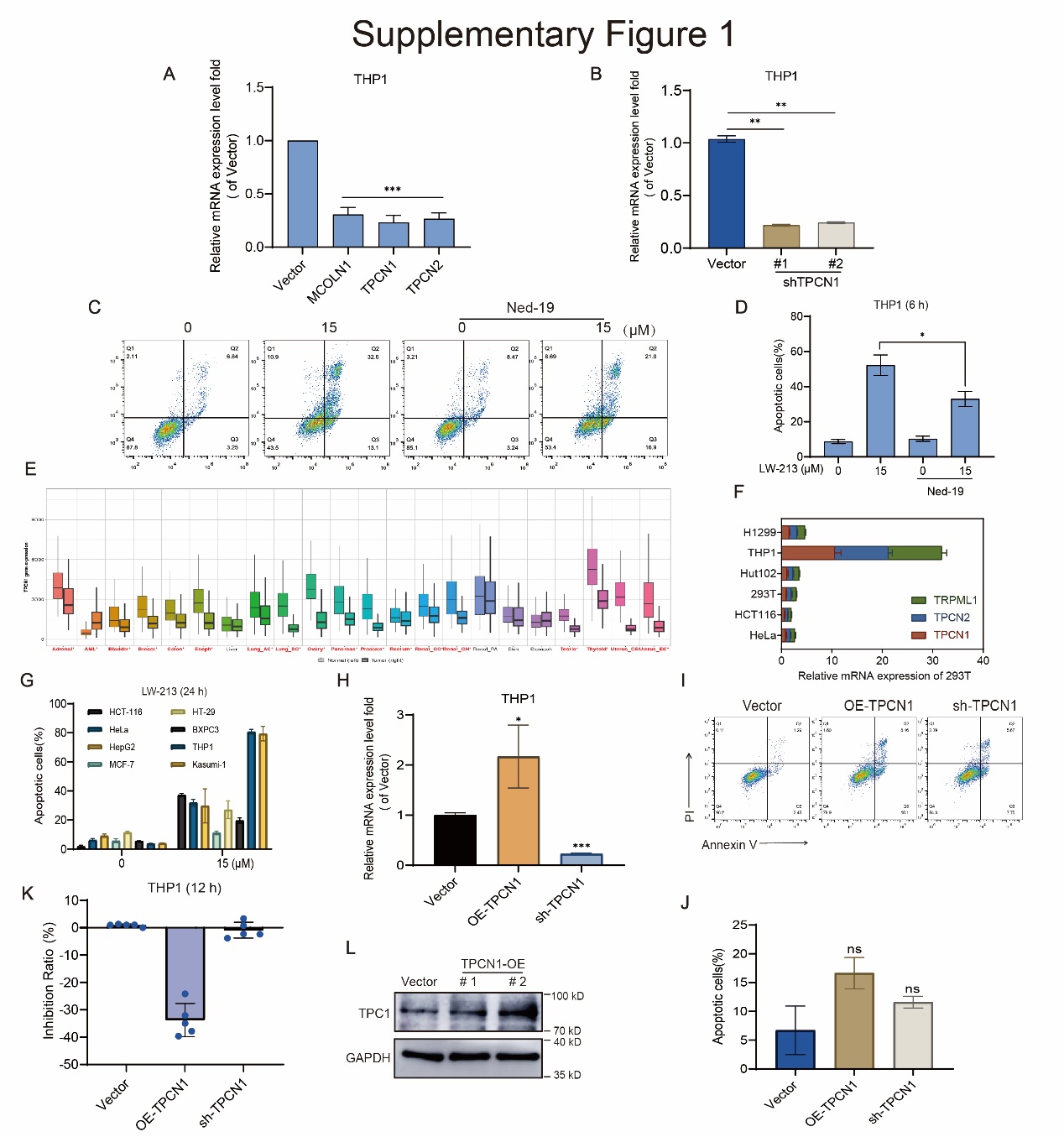


**Supplementary Figure 1**

(A) Total RNA was extracted to verify the knockdown efficiency of MCOLN1, TPCN1 and TPCN2 genes in THP1 cells. ^***^*p* < 0.001 compared to Vector group.

(B) Total RNA was extracted to verify the knockdown efficiency of TPCN1(#1, #2) genes in THP1 cells. ^**^*p* < 0.01 compared to Vector group.

(C-D) Flow cytometric analysis of Annexin V-FITC/PI-PerCP-stained cell line of THP1 cells were treated with 15 μM of LW-213 with/without Ned-19 (10 μM) for 6 hours. ^*^*p* < 0.05 compared to LW-213 (15 μM) group.

(E) TCGA database was used to analyze the expression of TPCN1 gene in tumor tissues and normal tissues.

(F) Total RNA was extracted to verify the mRNA level of MCOLN1, TPCN1 and TPCN2 genes in different cells.

(G) Flow cytometric analysis of Annexin V-FITC/PI-PerCP-stained cell line of different tumor cells were treated with 15 μM of LW-213 for 24 hours.

(H) Total RNA was extracted to verify the transfected efficiency of TPCN1 gene in THP1 cells .** p < 0.05, *** p < 0.001* compared to Vector group .

(I-J) Flow cytometric analysis of Annexin V-FITC/PI-PerCP-stained cell line of Vector, OE-TPCN1, sh-TPCN1 THP1 cells were treated with 15 μM of LW-213 for 12 hours.

(K) The growth inhibition effect on Vector, OE-TPCN1, and sh-TPCN1 THP1 cells were assessed by CCK8 assay at 12 hours. ns compared to Vector group.

(L) Total proteins were extracted to verify the overexpression efficiency of TPCN1 gene in HeLa cells.

Data are shown as Mean ± S.E.M. from three independent experiments. ^*^*p* < 0.05, ^**^*p* < 0.01, ^***^*p* < 0.001, ns indicates non-significant.


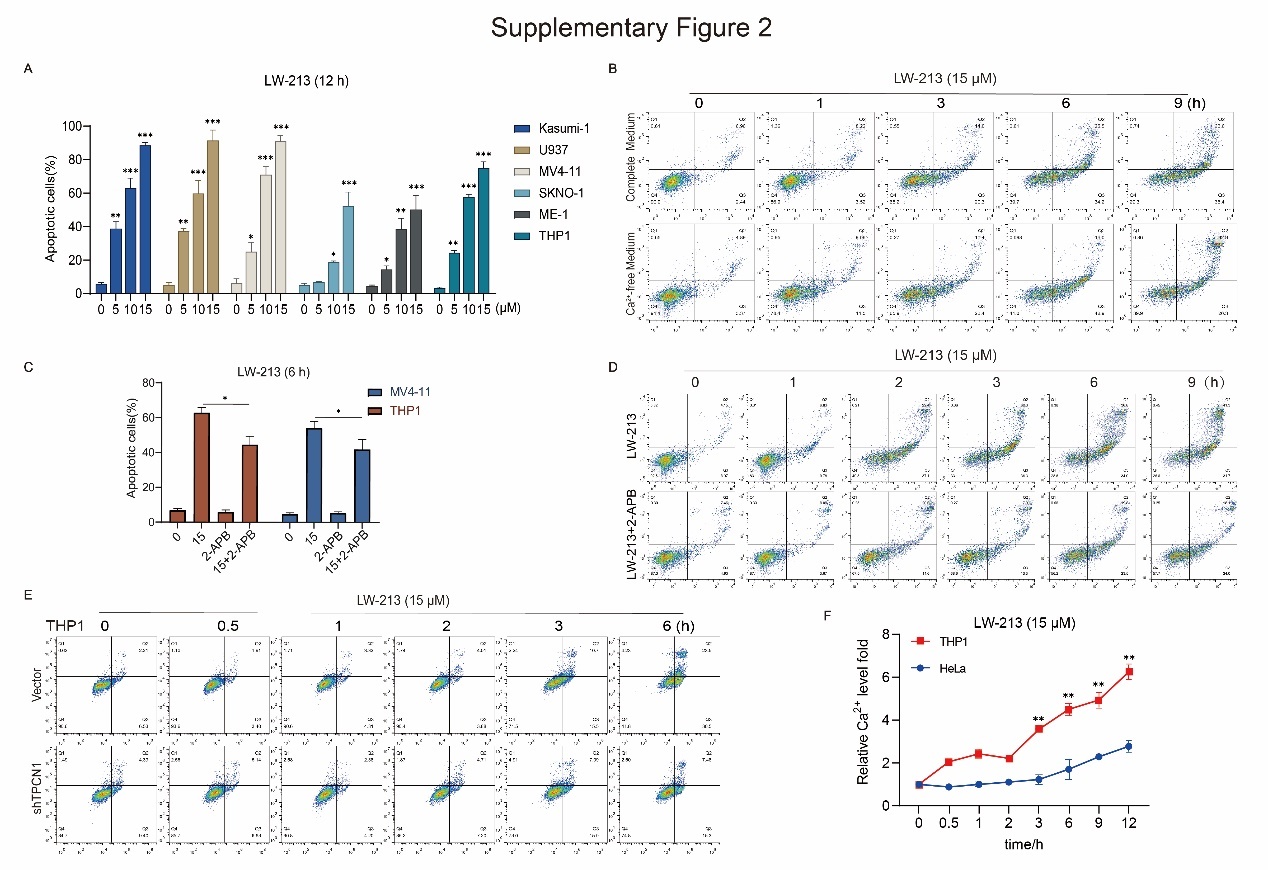


**Supplementary Figure 2**

(A) Flow cytometric analysis of Annexin V-FITC/PI-PerCP-stained cell lines of Kasumi-1, U937, MV4-11, SKNO-1, ME-1 and THP1 treated with 15 μM of LW-213 for 12 hours. ^*^*p* < 0.05, ^**^*p* < 0.01, ^***^*p* < 0.001 compared to LW-213 (0 μM) group.

(B) Flow cytometric analysis of Annexin V-FITC/PI-PerCP-stained cell line of THP1 was treated with 15 μM of LW-213 in complete medium and Ca^2+^ -free medium for 1, 3, 6, 9 hours.

(C) Flow cytometric analysis of Annexin V-FITC/PI-PerCP-stained cell line of THP1 and MV4-11 were treated with 15 μM of LW-213 with/without 2-APB (100 μM) for 6 hours. ^*^*p* < 0.05 compared to LW-213 (15 μM) group.

(D) Flow cytometric analysis of Annexin V-FITC/PI-PerCP-stained cell line of THP1 was treated with 15 μM of LW-213 with/without 2-APB (100 μM) for 1, 2, 3, 6, 9 hours.

(E) Flow cytometric analysis of Annexin V-FITC/PI-PerCP-stained cell line of Vector and sh-TPCN1 THP1 cells were treated with 15μM of LW-213 for 1, 2, 3, 6, 9 hours.

(F) The THP1 and HeLa cells were treated with 15 μM of LW-213 for 0.5, 1, 2, 3, 6, 9, 12 hours, respectively. The Ca^2+^ indicator Fluo3-AM measured cytoplasmic Ca^2+^ levels in each group of cells .^**^*p* < 0.01 compared to LW-213 (0 h) group.

Data are shown as Mean ± S.E.M. from three independent experiments. ^*^*p* < 0.05, ^**^*p* < 0.01, ^***^*p* < 0.001, ns indicates non-significant.


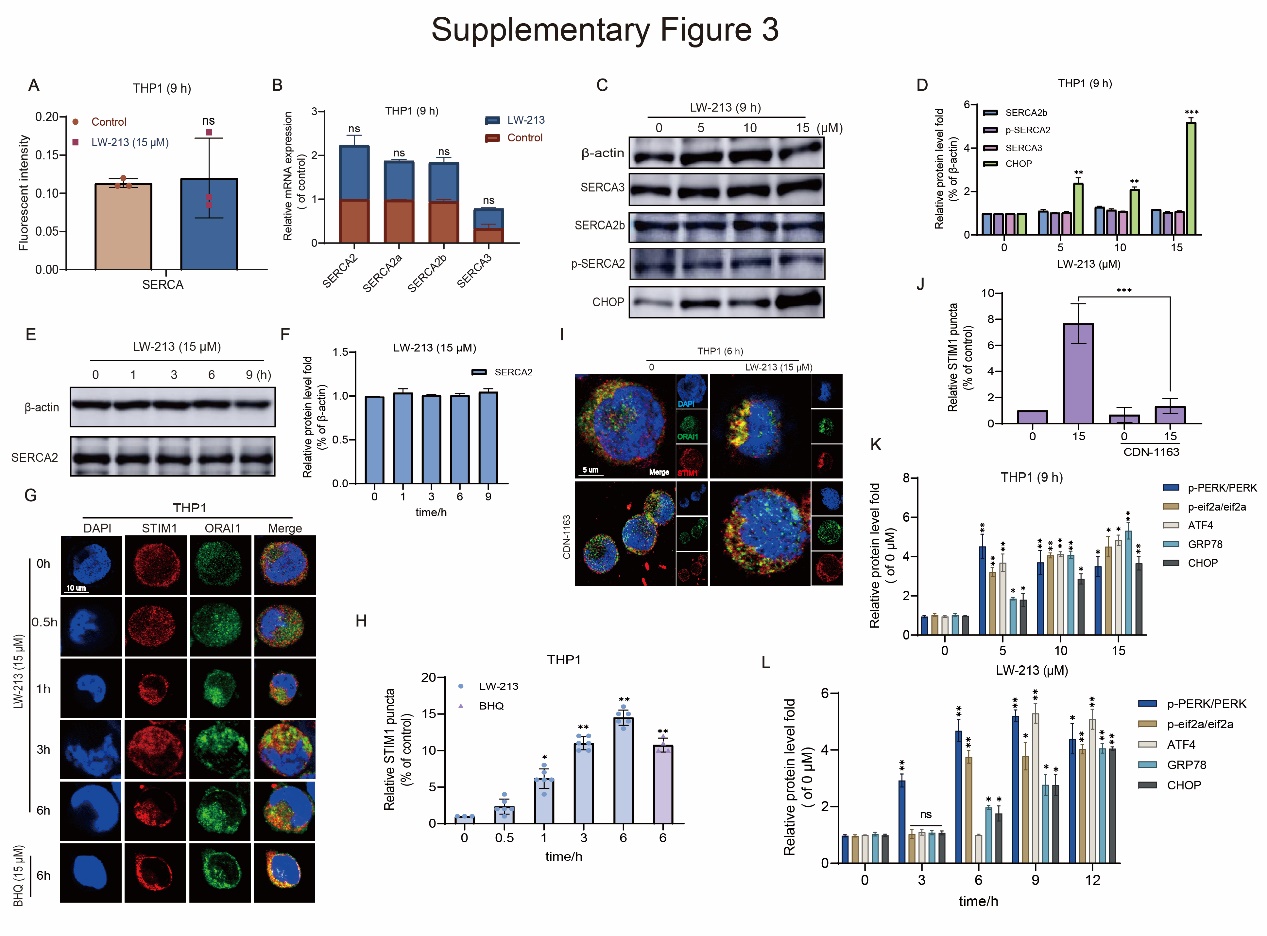


**Supplementary Figure 3**

(A) The THP1 cells were treated with 15 μM of LW-213 for 9 hours. Cells were collected to detect SECRA activity by *pNPP* method. ns compared to Control group.

(B) The THP1 cells were treated with 15 μM of LW-213 for 9 hours. Total RNA was extracted to verify the SERCA gene expression in THP1 cells. ns compared to Control group.

(C-D) The THP1 cells were exposed to LW-213 (5, 10, 15 μM) for 9 hours. ^**^*p* < 0.01, ^***^*p* < 0.001 compared to LW-213 (0 μM) group.

(E-F) The THP1 cells were exposed to LW-213 (15 μM) for 1, 3, 6, 9 hours. ns compared to LW-213 (0 h) group.

(G-H) The THP1 cells were treated with 15μM of LW-213 for 0.5, 1, 3, 6 hours and BHQ (15 μM) for 6 hours. Cells were collected and crawled for immunofluorescence staining of cell nuclei for DAPI (blue), STIM1 (red) and ORAI1 (green). They were detected by confocal microscopy (FV1000; Olympus) with FV10-ASW2.1 acquisition software (Olympus) at room temperature (original magnification × 1000; immersion objective × 100 × 40 with immersion oil type) (total cells in each group >100). ^*^*p* < 0.05, ^**^*p* < 0.01 compared to LW-213 (0 h) group.

(I-J) The THP1 cells were treated with 15 μM of LW-213 with/without CDN-1163 (50 μM) for 6 hours. Cells were collected and crawled for immunofluorescence staining of cell nuclei for DAPI (blue), STIM1 (red) and ORAI1 (green). They were detected by confocal microscopy (FV1000; Olympus) with FV10-ASW2.1 acquisition software (Olympus) at room temperature (original magnification × 1000; immersion objective × 100 × 40 with immersion oil type) (total cells in each group >100). ^***^*p* < 0.001 compared to LW-213 (15 μM) group.

(K-L) The ERS proteins blots were quantified using GraphPad Prism 9.0 software. Data are representative of 3 independent experiments. ^*^*p* < 0.05, ^**^*p* < 0.01, ns compared to LW-213 (0μM or 0h) group.

Data are shown as Mean ± S.E.M. from three independent experiments. ^*^*p* < 0.05, ^**^*p* < 0.01, ^***^*p* < 0.001, ns indicates non-significant.


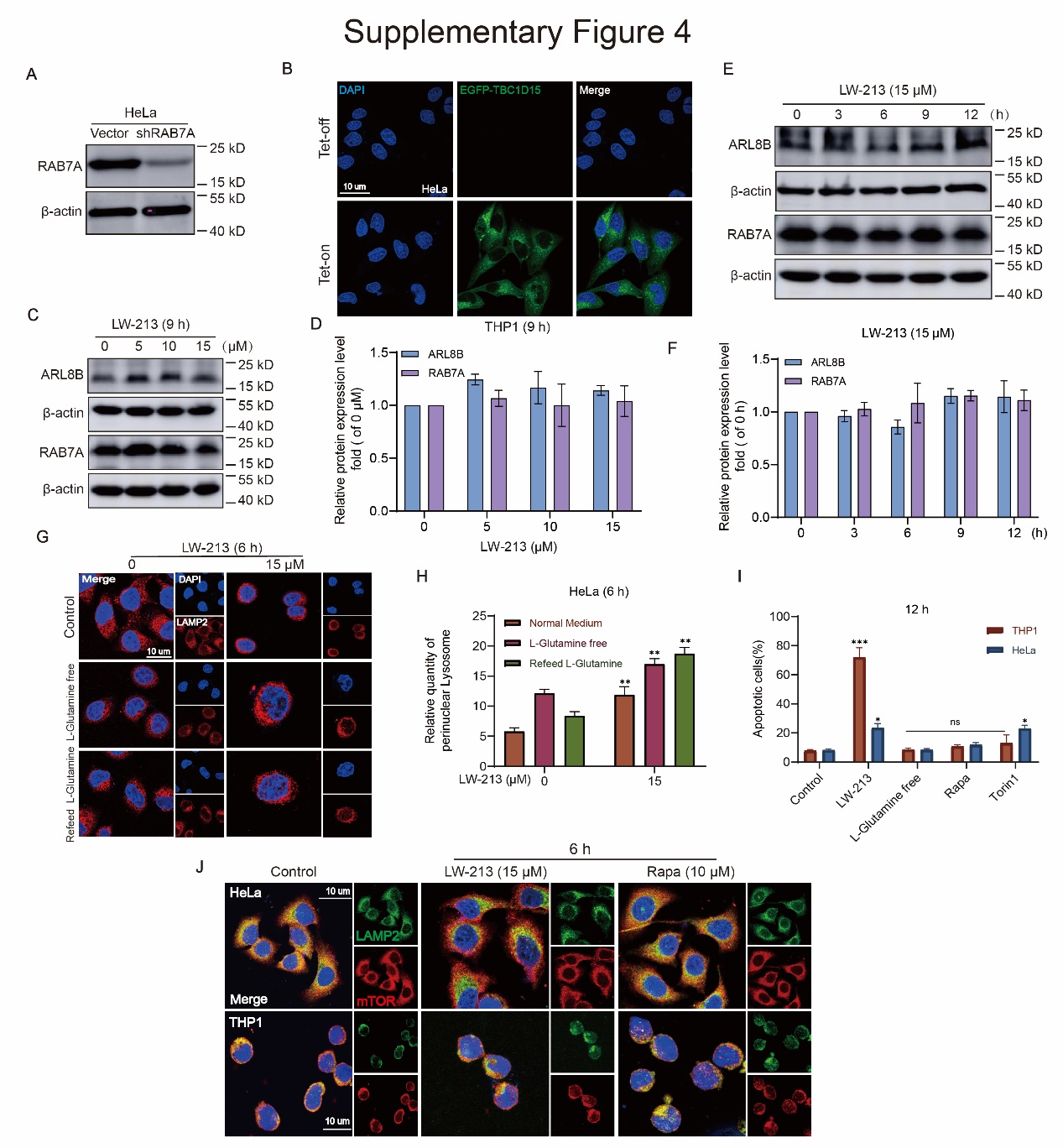


**Supplementary Figure 4**

(A) Total proteins were extracted to verify the knockdown efficiency of RAB7A gene in HeLa cells.

(B) HeLa cells were transfected empty vector and EGFP-TBC1D15 overexpression plasmid. The addition or withdrawal of 5 μM tetracycline for 24 hours could regulate the increase or recovery of TBC1D15 expression. Cells were collected and crawled for immunofluorescence staining of cell nuclei for DAPI (blue) and TBC1D15 (EGFP-green) (total cells in each group >100). They were detected by confocal microscopy (FV1000; Olympus) with FV10-ASW2.1 acquisition software (Olympus) at room temperature (original magnification × 1000; immersion objective × 100 with immersion oil type).

(C-F) Total proteins were extracted to verify RAB7A and ARL8B in THP1 cells.

(G-H) HeLa cells were treated with 15 μM of LW-213 without L-Glutamine and refeed L-Glutamine for 6 hours. Cells were collected and crawled for immunofluorescence staining of cell nuclei for DAPI (blue) and LAMP2 (red) (total cells in each group >100). They were detected by confocal microscopy (FV1000; Olympus) with FV10-ASW2.1 acquisition software (Olympus) at room temperature (original magnification × 1000; immersion objective × 100 with immersion oil type). ^**^*p* < 0.01compared to LW-213 (0 μM) group.

(I) Flow cytometric analysis of Annexin V-FITC/PI-PerCP-stained cell line of THP1 and HeLa cells were treated with LW-213 (15 μM), L-Glutamine-free, Rapa (10 μM) and Torin1(10 μM) for 12 hours. ^***^*p* < 0.001, ns compared to Control group.

(J) HeLa and THP1 cells were treated with LW-213 (15 μM) and Rapa (10 μM) for 6 hours, respectively. Cells were collected and crawled for immunofluorescence staining of cell nuclei for DAPI (blue), mTOR (red) and LAMP2 (green) (total cells in each group >100). They were detected by confocal microscopy (FV1000; Olympus) with FV10-ASW2.1 acquisition software (Olympus) at room temperature (original magnification × 1000; immersion objective × 100 with immersion oil type).

Data are shown as Mean ± S.E.M. from three independent experiments. ^*^*p* < 0.05, ^**^*p* < 0.01, ^***^*p* < 0.001, ns indicates non-significant.


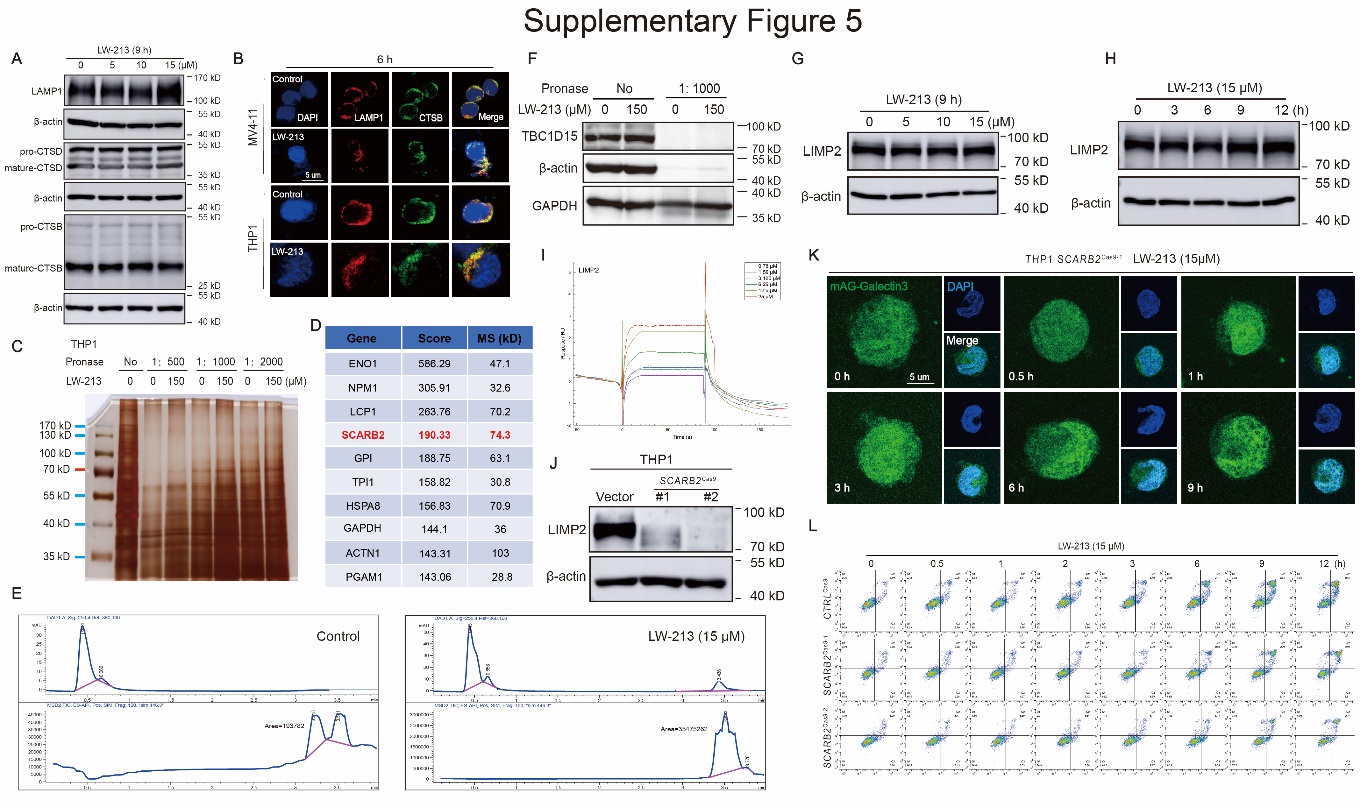


**Supplementary Figure 5**

(A) The THP1 cells were exposed to LW-213 (5, 10, 15 μM) for 9 hours.

(B) MV4-11 and THP1 cells were treated with LW-213 (15 μM) for 6 hours, respectively. Cells were collected and crawled for immunofluorescence staining of cell nuclei for DAPI (blue), LAMP1 (red) and CTSB (green) (total cells in each group >100). They were detected by confocal microscopy (FV1000; Olympus) with FV10-ASW2.1 acquisition software (Olympus) at room temperature (original magnification × 1000; immersion objective × 100 with immersion oil type).

(C-D) The remaining protein components after Pronase (20 mg/mL, volume ratio 1:1000) enzymatic hydrolysis were analyzed by LC-MS.

(E) The contents of LW-213 in lysosomes and cytoplasm were analyzed by HPLC.

(F) Western Blot analysis of TBC1D15 in THP1 treated by 150μM LW-213 and Pronase (20 mg/mL, volume ratio 1:1000).

(G) The THP1 cells were exposed to LW-213 (5, 10, 15 μM) for 9 hours.

(H) The THP1 cells were exposed to LW-213 (15 μM) for 3, 6, 9, 12 hours.

(I) The affinity of LIMP2 to LW-213 at different concentrations was detected by SPR technique.

(J) Total proteins were extracted to verify the knockout efficiency of SCARB2 gene in THP1 cells.

(K) THP1 cells were transfected mAG-Galectin3 plasmid, were exposed to LW-213 (15 μM) for 0.5, 1, 3, 6, 9 hours. Cells were collected and crawled for immunofluorescence staining of cell nuclei for DAPI (blue) and mAG-Galectin3 (green) (total cells in each group >100). They were detected by confocal microscopy (FV1000; Olympus) with FV10-ASW2.1 acquisition software (Olympus) at room temperature (original magnification × 1000; immersion objective × 100 with immersion oil type).

(L) Flow cytometric analysis of Annexin V-FITC/PI-PerCP-stained cell line of Vector and LIMP2-KO (#1, #2) THP1 cells were treated with LW-213 (15 μM) for 0.5, 1, 2, 3, 6, 9 12 hours.

Data are shown as Mean ± S.E.M. from three independent experiments. ^*^*p* < 0.05, ^**^*p* < 0.01, ^***^*p* < 0.001, ns indicates non-significant.


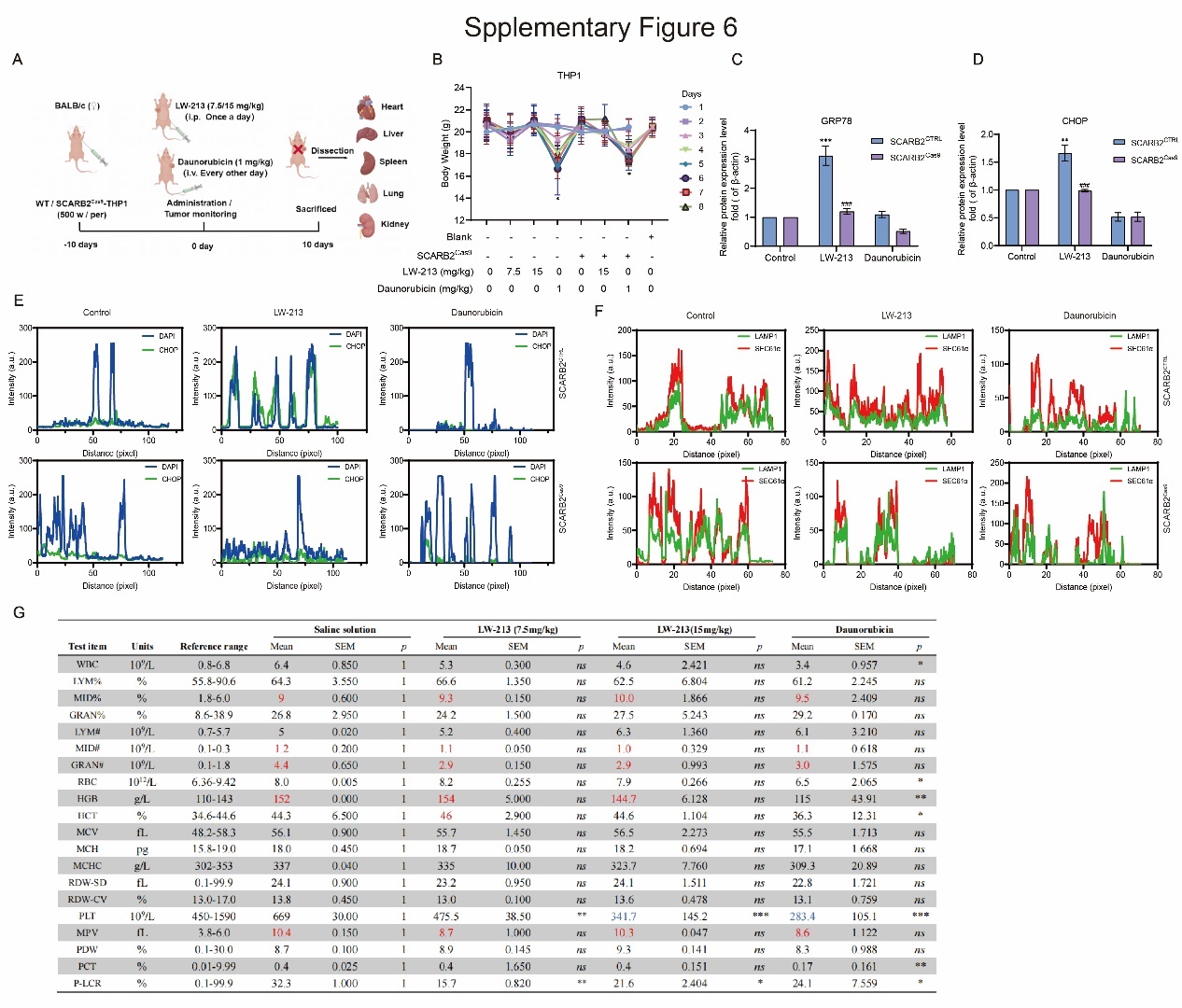


**Supplementary Figure 6**

(A) The flow diagram illustrated the *BALB/c* nude mice model of THP1 cell xenograft.

(B) The body weight of THP1 or KO-LIMP2 THP1 cells bearing *BALB/c* nude mice after treatment by LW-213 (7.5, 15 mg/kg) and Daunorubicin (1 mg/kg) group. ^*^*p* < 0.05 compared to Daunorubicin (1 d) group.

(C-D) The protein blots were quantified using GraphPad Prism 9.0 software. Data are representative of 3 independent experiments.  ^***^*p* < 0.001 compared to Control group, ^###^*p* < 0.001 compared to SCARB2^CTRL^ group.

(E-F) The IF was quantified using Image J. Data are representative of 3 independent experiments.

(G) The blood routine analysis of ICR mice with saline, LW-213 or Daunorubicin treatment.

Data are shown as Mean ± S.E.M. from three independent experiments. ^*^*p* < 0.05, ^**^*p* < 0.01, ^***^*p* < 0.001, ns indicates non-significant.

**Supplementary Video 1**

The THP1 cells, preloaded with the Fluo3-AM (1:1000) fluorescent probe, were cultured in laser confocal microscope dish at a density of 3000 cells per well. During the treatment of THP1 cells with LW-213 (15 μM) for 9 hours, the change in green fluorescence intensity was dynamically monitored using a live cell workstation. They were detected by spinning disk confocal microscope Andor BC43 (Oxford Instrument, Abingdon, UK) at 37℃, 5% CO2 (original magnification × 400; immersion objective × 20). Images are representative of 3 independent experiments.

**Supplementary Video 2**

The THP1 cells, preloaded with the ER-tracker (red) and Lysotracker (green) (1:1000) fluorescent probes, were cultured in laser confocal microscope dish at a density of 3000 cells per well. During the treatment of THP1 cells with LW-213 (15 μM) for 9 hours, the change in spatial position of ER and lysosome was dynamically observed using a live cell workstation. They were detected by spinning disk confocal microscope Andor BC43 (Oxford Instrument, Abingdon, UK) at 37℃, 5% CO2 (original magnification × 400; immersion objective × 40). Images are representative of 3 independent experiments.

**Supplementary Table 1**

**
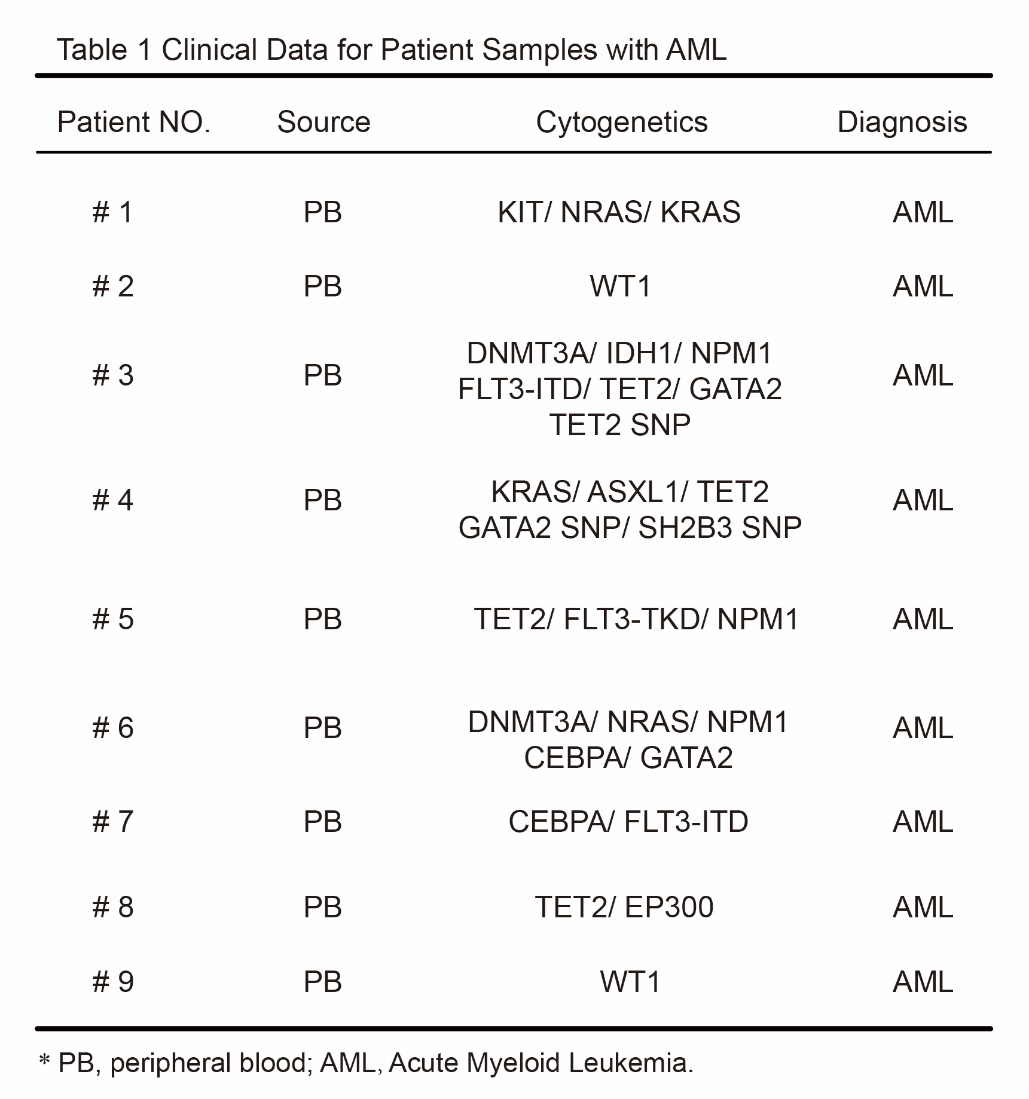
**

**Graphic Abstract**

**
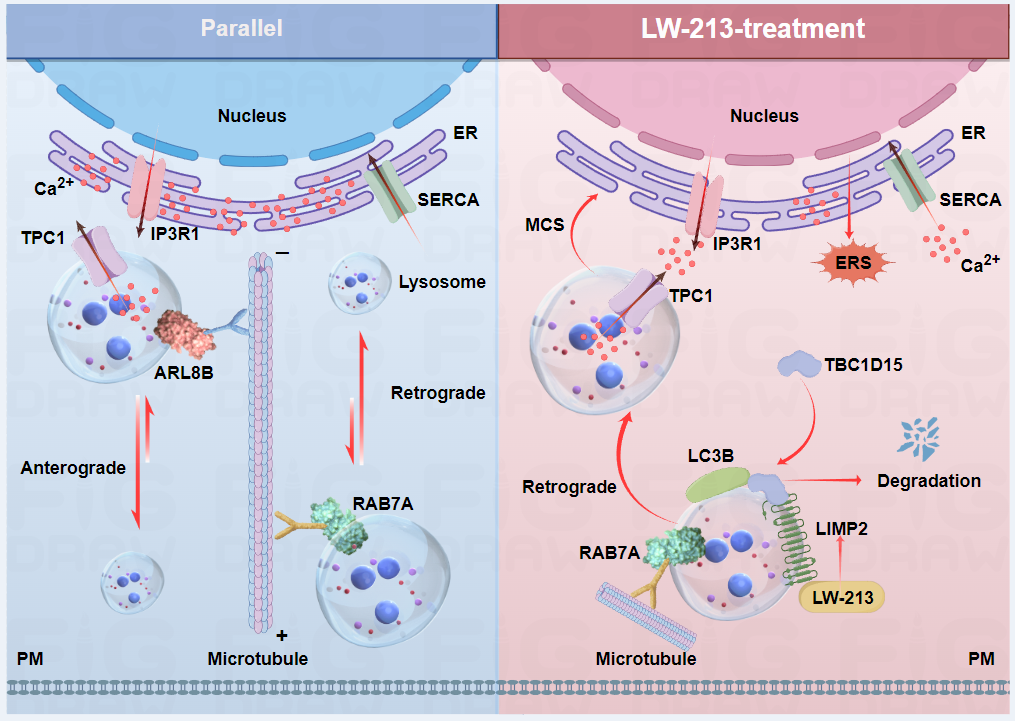
**
