# Supplementary figures and images for: Activation of Lysosomal Retrograde Transport Triggers TPC1‐IP3R1 Ca2+ Crosstalk at Lysosome‐ER MCSs Leading to Lethal Depleting of ER Calcium
Source: Adv Sci (Weinh). 2025 Jul 25;12(39):e15313. doi: 10.1002/advs.202415313 (PMC12533322; doi:10.1002/advs.202415313)

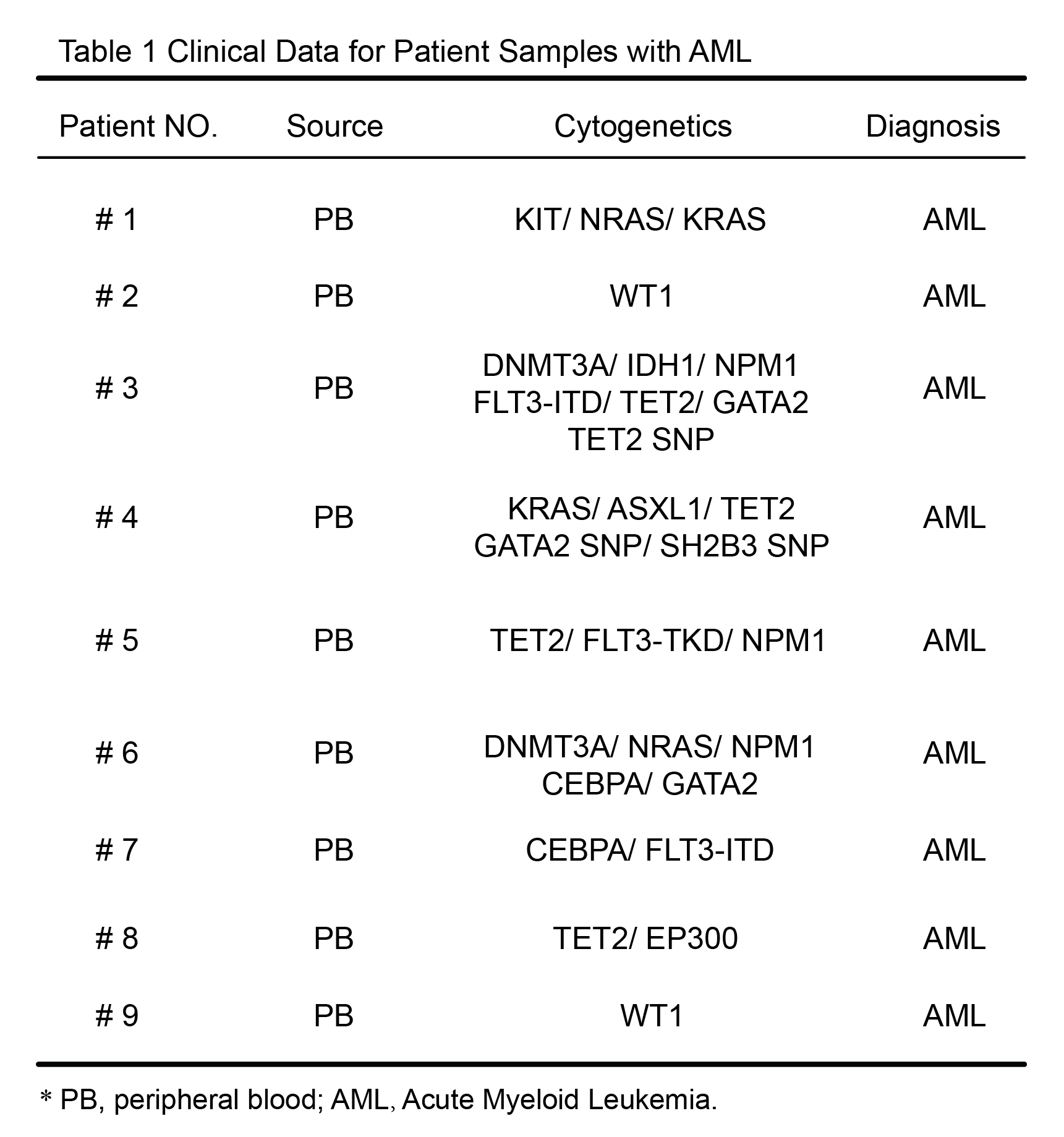

Supplement: Supplementary file 6 — Supplemental Table [file ADVS-12-e15313-s002.jpg]
